# Association of remnant cholesterol with frailty: findings from observational and Mendelian randomization analyses

**DOI:** 10.1186/s12944-023-01882-4

**Published:** 2023-08-03

**Authors:** Yuanlong Hu, Xiaojie Wang, Lin Lin, Jiaming Huan, Yuan Li, Lei Zhang, Yunlun Li

**Affiliations:** 1 First Clinical Medical College, Shandong University of Traditional Chinese Medicine, Jinan, Shandong China; 2 Shandong Province Engineering Laboratory of Traditional Chinese Medicine Precise Diagnosis and Treatment of Cardiovascular Disease, Shandong University of Traditional Chinese Medicine, Jinan, Shandong China; 3 Faculty of Chinese Medicine, Macau University of Science and Technology, Taipa, Macau, China; 4 Innovative Institute of Chinese Medicine and Pharmacy, Shandong University of Traditional Chinese Medicine, Jinan, China; 5 Experimental Center, Shandong University of Traditional Chinese Medicine, Jinan, Shandong China; 6 Key Laboratory of Traditional Chinese Medicine Classical Theory, Ministry of Education, Shandong University of Traditional Chinese Medicine, Jinan, Shandong China; 7 Shandong Provincial Key Laboratory of Traditional Chinese Medicine for Basic Research, Shandong University of Traditional Chinese Medicine, Jinan, Shandong China; 8 College of Traditional Chinese Medicine, Shandong University of Traditional Chinese Medicine, Jinan, China; 9 Department of Cardiovascular, Affiliated Hospital of Shandong University of Traditional Chinese Medicine, Jinan, Shandong China

**Keywords:** Frailty, Remnant cholesterol, Aging, Mendelian randomization

## Abstract

**Background:**

Recent insights suggest that remnant cholesterol (RC) plays a role in cellular senescence, yet its specific contribution to frailty remains indeterminate. Through the integration of observational and mendelian randomization (MR) studies, this research explores the impact of elevated serum RC levels on frailty susceptibility.

**Methods:**

A dual-method approach, combining an observational study with an MR study, was employed to investigate the connection between RC and frailty. The observational study included 11,838 participants from the National Health and Nutrition Examination Survey. Multivariable logistic regression and propensity score matching were employed to control for potential confounders. The non-linear relationship was assessed using restricted cubic splines. To circumvent observational study limitations, a two-sample MR analysis was conducted using the inverse-variance weighted method, leveraging genome-wide association studies (GWAS) data.

**Results:**

After adjusting for potential confounding variables, the observational study identified a significant association between high serum RC levels and frailty in middle-aged and older adults (odds ratio [OR] = 1.67, 95% confidence interval [CI] = 1.20 to 2.33, *P* = 0.003), exhibiting a non-linear dose–response correlation (non-linear *P* = 0.011). This association persisted after propensity score matching (OR = 1.53, 95% CI = 1.14 to 2.06, *P* = 0.005). The MR study echoed these results, demonstrating a causal association of RC with the frailty index (β = 0.059, 95% CI = 0.033 to 0.085, *P* = 1.05E-05), consistent with the observational findings (β = 0.017, 95% CI = 0.008 to 0.026, *P* = 4.51E-04).

**Conclusion:**

This study provides evidence that higher RC levels amplify frailty risk in middle-aged and older adults, implying that the reduction of RC levels may present a promising strategy for frailty prevention and management.

**Supplementary Information:**

The online version contains supplementary material available at 10.1186/s12944-023-01882-4.

## Introduction

With advancing age, individuals tend to experience a progressive accumulation of health-related deficits, which eventually leads to cumulating in a state of frailty. This clinical condition, characterized by vulnerability, signifies severe dysregulation within a biologically complex dynamical system inherent to the aging process [1–3]. Epidemiological evidence underscores the high prevalence of frailty among the elderly demographic. For instance, a comprehensive meta-analysis incorporating 57 studies revealed that frailty affects approximately 26.8% of the aging population [4]. Given the heightened predisposition of this demographic to adverse clinical outcomes, there is a growing emphasis on the early identification and modification of risk factors related to frailty.

In the elderly population, frailty is associated with a significantly increased risk for the development of cardiovascular disease (CVD) and the occurrence of major adverse cardiovascular events [5, 6]. Evidence from a Mendelian randomization (MR) study underscores a bidirectional causal relationship between frailty and coronary heart disease (CHD) [7]. This correlation could stem from shared risk factors [8], especially the elevated serum level of cholesterol. Previous research has indicated a correlation between increased serum levels of low-density lipoprotein cholesterol (LDL-C) and the risk of frailty[9]. Numerous researches in recent years have identified remnant cholesterol (RC) as an independent risk factor that contributes to the occurrence of incident cardiovascular events [10–12]. RC is computed as the difference between total cholesterol (TC) and the aggregate of high-density lipoprotein cholesterol (HDL-C) and LDL-C, primarily representing the cholesterol content of a subset of triglyceride-rich lipoproteins (TRLs) [13]. One proposed mechanism suggests that hydrolyzed products from TRLs may expedite cellular senescence in a range of cells, including endothelial cells, vascular smooth muscle cells, macrophages, and adipose-derived mesenchymal stem cells (AMSC) [14]. While direct evidence is available for AMSC, supporting evidence for other cell types remains largely indirect [15]. Cellular senescence at the cellular level is a crucial mechanism driving frailty [16]. However, the exact mechanism through which RC is associated with the risk of frailty remains to be elucidated.

The aim of this study was to examine the potential association between RC and frailty through two distinct, yet complementary approaches. The initial phase of the investigation involved an observational study using data from the National Health and Nutrition Examination Survey (NHANES) to assess the association. However, acknowledging the limitations of observational studies, primarily the prevalence of confounding factors and potential for reverse causality, a MR study was also employed. In the MR study, genetic variants that influence serum remnant cholesterol levels were utilized as instrumental variables, simulating the conditions of a randomized experiment. This technique leverages the natural random distribution of genetic variants during gamete formation and conception, thereby effectively mitigating confounding elements and the risk of reverse causality. Such an approach provides a more robust evidence base supporting any potential causal link between RC and frailty [17].

## Materials and methods

### Study population for the observational epidemiological study

The present observational study leveraged data across eight NHANES cycles spanning from 2003–2004 through 2017–2018. The NHANES is a nationally representative survey dedicated to assessing the health and nutritional condition of both adult and pediatric populations in the United States [18]. The inclusion criteria for this study specified non-institutionalized individuals aged 40 years and above who had undergone lipid profiling. Subjects with triglyceride levels equal to or exceeding 400 mg/dl were excluded from the study. Protocols #98–12, #2005–06, #2011–17, and #2018–01 were granted approval by the Institutional Review Board of the National Center for Health Statistics (NCHS) [19].

### Measurement of variables in the observational epidemiological study

For this observational epidemiological study, the primary exposure variables comprised of RC, RC-to-TC ratio, and TC-to-LDL-C ratio. RC was deduced by subtracting HDL-C and LDL-C from TC. As LDL-C direct measurements were not provided by NHANES, its levels for the primary analyses were calculated using the Martin-Hopkins equation [20]. A preceding study [21] demonstrated that the Martin-Hopkins equation provides more accurate estimations of LDL-C (for triglyceride levels of < 400 mg/dl) compared to the Friedewald [22] and Sampson equations [23]. For comparison, LDL-C values were also computed using the Friedewald and Sampson equations.

Frailty status, defined by the frailty index (FI), was identified as the primary outcome measure. FI was determined using 49 accessible items, based on the deficit accumulation approach proposed by Rockwood et al. (Supplementary Table 1) [24]. FI was computed by dividing the sum of deficits by the total number of items, yielding a score between 0 and 1. Participants possessing an FI greater than 0.21 were classified as frail [25, 26]. Concurrently, the Fried frailty phenotype (FP) was assessed according to a formerly published method [27]; meeting at least three criteria was deemed indicative of frailty [3].

The study accounted for various covariates, including sociodemographic attributes, socioeconomic status, lifestyle behaviors, frailty-associated risk factors, clinical comorbidities, and current medications. The sociodemographic variables included age, gender, ethnicity, education level, and marital status. Household income as a percentage of the federal poverty level (FPL) was utilized to gauge the socioeconomic status, categorized as poor (≤ 100% FPL), near poor (101–200% FPL), or non-poor (> 200% FPL). The healthy eating index—2015 (HEI-2015) was employed to evaluate dietary behaviors, which assesses adherence to the 2015–2020 Dietary Guidelines for Americans. The smoking status was segmented into three categories: never, former, or current. “Never smokers” were those who smoked fewer than 100 cigarettes throughout their lifetime. “Current smokers” referred to those who consumed more than 100 cigarettes in their lifetime and were still active smokers. “Former smokers” were those who ceased smoking after consuming more than 100 cigarettes. The study also included frailty-related risk factors such as body mass index (BMI), systolic blood pressure (SBP), diastolic blood pressure (DBP), and estimated glomerular filtration rate (eGFR), which was computed using creatinine-based eGFR (eGFR_Cr_) via the CKD-EPI (CKD Epidemiology Collaboration) equations [28]. Self-reported histories of cardiovascular disease (CVD) and Type 2 diabetes mellitus (T2DM) were counted as clinical comorbidities. CVD was defined as self-reported instances of CHD, congestive heart failure (HF), heart attacks, strokes, or angina. The study also accounted for covariates, which included information on the current usage of specific medications, such as statins, anti-diabetic drugs, and anti-hypertensive drugs.

### Data Source for mendelian randomization study

In the MR analyses, summary-level data from large-scale genome-wide association studies (GWAS) were utilized to assess the potential causal influence of RC on frailty. The GWAS summary data for RC was procured from the UK Biobank, encompassing data from 115,082 participants [29]. The quantification of remnant cholesterol was computed as the TC minus the sum of LDL-C and HDL-C. The TC, LDL-C, and HDL-C values were acquired through high-throughput nuclear magnetic resonance metabolomics conducted by Nightingale Health (biomarker quantification version 2020) [29]. The data were subsequently adjusted for variables such as age, sex, fasting status, and the genotyping array.

The GWAS summary data for the FI was derived from a meta-analysis of UK Biobank participants of European descent (*N* = 164,610) and Swedish TwinGene participants (*N* = 10,616) [30]. The UK Biobank participants were aged between 60 to 70 years, and the Swedish TwinGene participants ranged from 41 to 87 years. During the GWAS meta-analysis, covariate adjustments were made for age, sex, assessment center, and the genotyping array. The GWAS summary data for the FP was obtained from the study conducted by Ye et al., involving 386,565 individuals of European ancestry from the UK Biobank [31]. The GWAS summary statistics are compiled in Supplementary Table 2.

### Selection of genetic instruments

This study entailed the selection of single nucleotide polymorphisms (SNPs) that exhibited a significant association with RC, FI, or FP (*P* < 5 × 10^−8^), and independent segregation (R^2^ < 0.001, within a 5000 kb window), with no evidence of linkage disequilibrium (LD). A clumping algorithm employed, referencing the 1000 genomes panel, to identify and exclude SNPs displaying LD. Furthermore, SNPs demonstrating palindrome alleles (A/T or G/C), which could potentially lead to strand ambiguity issues, were excluded from the study. The F-statistic was computed using a formula outlined in prior research, and SNPs demonstrating an F-statistic exceeding 10 were deemed as strong genetic instrumental variables (IVs) for RC level, consistent with the Staiger-Stock rule [32, 33].

### Statistical analysis of observational epidemiological study

All analyses were performed using sampling weights, strata, and primary sampling units to ensure the derivation of nationally representative estimates. To circumvent the reduction in sample size attributable to missing covariate data, these gaps were filled in using the missForest method within the missForest R package. Spearman rank correlation coefficient tests were conducted to analyze the correlation between LDL-C values as calculated by different formulas.

Logistic regression analyses were employed to determine the association between RC, RC-to-TC ratio, RC-to-LDL-C ratio, and the likelihood of frailty. Further, FI, treated as a continuous outcome variable, was included in the linear regression model for subsequent re-analysis. We utilized two different methods to correct for the influence of confounding factors. The presence of multicollinearity was verified using the generalized variance inflation factor (GVIF), with variables exhibiting a GVIF > 10 excluded from the model. Three models were fitted in a stepwise manner. Model 1 adjusted for age (continuous), gender (female and male), ethnicity (white, black, or other), education level (below high school, or high school and above), family income (poor, near poor, or non-poor), and marital status (non-married or married). Model 2 accommodated the variables in model 1 and also adjusted for smoking status (never, former, or current) and HEI-2015 (quartile). Model 3 incorporated the variables from model 2 and further adjusted for BMI (continuous), SBP (continuous), DBP (continuous), eGFR level (≥ 90, 60 to 89, and < 60 mL/min per 1.73 m^2^), CVD (no or yes), T2DM (no or yes), statins use (no or yes), anti-diabetic drug use (no or yes), and anti-hypertensive drug use (no or yes). For RC, model 4 was created, which included TC (continuous) and LDL-C (continuous) adjustments in addition to the variables in model 3. Secondly, a 1:1 propensity score matching (PSM) method was employed to control for potential confounding variables, considering all variables in model 3 and sampling weights. The nearest-neighbor matching was conducted within a caliper of 0.05 on the propensity score scale, using the MatchIt R package. Lastly, to model a potential dose–response relationship of RC with frailty, the restricted cubic splines (RCS) with three knots were applied.

The relationship between RC, the RC-to-TC ratio, and the RC-to-LDL-C ratio with the likelihood of frailty was scrutinized in various demographic subgroups. These were divided by age (< 60 and ≥ 60 years), gender (female and male), BMI (< 30 and ≥ 30 kg/m^2^), eGFR level (≥ 90, 60 to 89, and < 60 mL/min per 1.73 m^2^), presence of CVD or T2DM (no or yes), and hypertension (no or yes) in the logistic models. Hypertension was defined as SBP ≥ 140 mmHg, DBP ≥ 90 mmHg, or current utilization of anti-hypertensive medications. The multiplicative interaction and the determination of effect size variations among different population subgroups were assessed via likelihood ratio tests.

Complementary sensitivity analyses were conducted. Firstly, the association of RC with frailty was re-evaluated excluding heart failure, coronary heart disease, angina, heart attack, stroke, and T2DM from the FI. Subsequently, differing strategies for managing missing values were employed for sensitivity analysis, encompassing direct deletion of missing values and multiple imputation. Ten complete datasets were synthesized via multiple imputation using the mice R package. Given the intricate sampling design, results were consolidated in accordance with Rubin’s rule, utilizing the survey and mitools R packages in R. Finally, the results generated using the Friedewald equation and Sampson equation were juxtaposed with the principal results.

All computations were conducted using R and RStudio software. To correct for the three tested null-hypotheses, Bonferroni adjustment was applied (Bonferroni: 0.05/3 = 0.017). A *P*-value < 0.017 was considered as being statistically significant.

### Mendelian randomization analysis

The causal effect of RC on the FI or FP was assessed employing the multiplicative random-effects inverse-variance weighted (IVW) method, undeterred by heterogeneity statistics. Additional MR methods, including weighted median, MR-Egger, simple median, and MR Pleiotropy RESidual Sum and Outlier (MR-PRESSO) were incorporated into the data analysis process. Reverse direction MR was conducted to evaluate any pre-existing reverse-direction causal association. The MR-Steiger directionality test was employed to validate causality directionality [34]. Multivariable MR, facilitated by the IVW method, was utilized to estimate the direct causal impact of RC on the FI, incorporating adjustments for TC, LDL-C, BMI, CHD, HF, stroke, T2DM, SBP, and DBP. The execution of these methods relied on the TwoSampleMR (version 0.5.6) [35] and MRPRESSO (version 1.0) R packages [36].

Three strategies were implemented to test for potential pleiotropy. First, the intercept test from MR-Egger regression served as the principal method to identify directional pleiotropy. The PhenoScanner web tool was utilized next, aiming to identify SNPs linked to potential confounders; such SNPs were then extracted from the IVs before re-analysis of the primary results [37]. The MR-PRESSO test was subsequently employed to identify and rectify horizontal pleiotropy through the MRPRESSO (version 1.0) R package [36]. Heterogeneity was quantified using the Cochran’s Q Statistic and I^2^ value, supported by visual assessment via funnel plot. After removing SNPs resulting in the heterogeneity, the main results were re-analyzed. After SNPs contributing to heterogeneity were removed, the primary results were revisited. The MRlap (version 0.0.3.0) R package was used to account for and rectify potential bias induced by sample overlap [38].

## Results

### Observational epidemiological analysis for association of RC with frailty

**Fig. 1:**
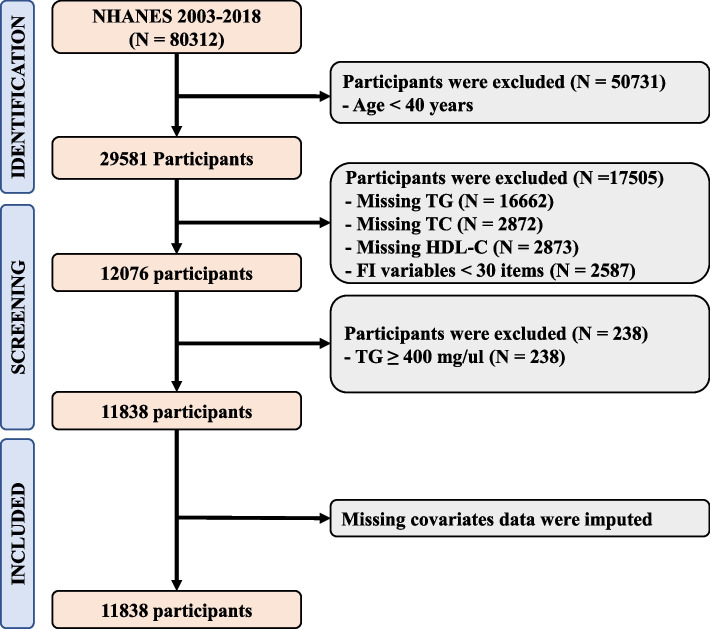
Flowchart of Participant Inclusion and Exclusion

The study incorporated a total of 11,838 participants as delineated in Fig. 1. Given the sampling design, this represents a potential sampling of 58.32 million. A summary of the population characteristics is presented in Table 1. The participants’ average age was 59 (standard deviation [SD] 11.90), with 52.43% (6,059) being female. The average FI was 0.160 with a standard deviation of 0.002, and 24.88% of participants were categorized as frail according to the FI. Frail participants, in comparison to non-frail participants, were found to be older, with a higher BMI, higher SBP, lower DBP, lower eGFR, lower HEI-2015, and had a higher prevalence of CVD or T2DM. Correlation tests using Spearman method identified a significant positive correlation between LDL-C as determined by the Martin-Hopkins equation and the Friedewald equation (Spearman correlation = 0.988, *P* < 2.2E-16), as well as the Sampson equation (Spearman correlation = 0.998, *P* < 2.2E-16), as depicted in Supplementary Fig. 1.

**Table 1:** Population characteristics across frailty status

Characteristics	Total (*N* = 17,052)	Non-Frailty (*N* = 13,233)	Frailty (*N* = 3819)	*P*-value
**Age,** years	59 (11.90)	57 (11.46)	63 (12.38)	2.05E-30
**Gender**				1.16E-18
Female	6059 (52.43%)	3973 (49.31%)	2086 (61.84%)	
Male	5779 (47.57%)	4282 (50.69%)	1497 (38.16%)	
**Ethnicity**				2.63E-13
White	5545 (73.78%)	3869 (75.30%)	1676 (69.17%)	
Black	2389 (9.60%)	1520 (8.18%)	869 (13.90%)	
Other	3904 (16.62%)	2866 (16.52%)	1038 (16.93%)	
**Education level**				2.66E-22
Below High School	3397 (17.66%)	2043 (14.39%)	1354 (27.54%)	
High School and above	8441 (82.34%)	6212 (85.61%)	2229 (72.46%)	
**Marital status**				2.49E-18
Non-Married	5056 (37.01%)	3143 (33.09%)	1913 (48.86%)	
Married	6782 (62.99%)	5112 (66.91%)	1670 (51.14%)	
**Family income**				8.30E-50
Poor	2012 (10.47%)	1108 (7.55%)	904 (19.27%)	
Near poor	3375 (19.96%)	2048 (16.06%)	1327 (31.75%)	
Non-poor	6451 (69.57%)	5099 (76.39%)	1352 (48.98%)	
**Smoking status**				2.33E-15
Never	5980 (50.35%)	4392 (53.18%)	1588 (41.78%)	
Former	3703 (31.48%)	2495 (30.78%)	1208 (33.61%)	
Current	2155 (18.17%)	1368 (16.04%)	787 (24.61%)	
**BMI**, kg/m^2^	29 (6.64)	29 (6.05)	31 (7.86)	1.81E-21
**SBP,** mmHg	126 (18.40)	125 (17.69)	129 (20.06)	1.41E-15
**DBP,** mmHg	70 (11.69)	71 (11.18)	68 (12.88)	7.33E-16
**HEI-2015**				1.07E-12
Quartile 1	2960 (26.04%)	1929 (24.80%)	1031 (29.78%)	
Quartile 2	2959 (24.67%)	1961 (23.65%)	998 (27.75%)	
Quartile 3	2959 (24.49%)	2108 (24.36%)	851 (24.89%)	
Quartile 4	2960 (24.80%)	2257 (27.20%)	703 (17.58%)	
**eGFR,** ml/min per 1.73 m^2^				4.68E-46
≥ 90	5104 (43.56%)	3895 (45.87%)	1209 (36.60%)	
60 to 89	5106 (45.58%)	3633 (47.14%)	1473 (40.90%)	
< 60	1628 (10.85%)	727 (7.00%)	901 (22.50%)	
**Type 2 DM**	2188 (13.96%)	883 (8.15%)	1305 (31.49%)	8.66E-49
**CVD**	2100 (14.75%)	692 (7.81%)	1408 (35.69%)	1.57E-52
**Statins use**	3296 (26.63%)	1830 (21.86%)	1466 (41.01%)	4.36E-26
**Anti-diabetic drug use**	2012 (13.20%)	837 (8.11%)	1175 (28.56%)	1.62E-43
**Anti-hypertensive drug use**	1122 (8.49%)	680 (7.45%)	442 (11.62%)	2.61E-08
**TG,** mg/dl	109 (76, 158)	105 (74, 153)	121 (86, 175)	1.54E-15
**TC,** mg/dl	197.94 (41.36)	200.41 (39.95)	190.49 (44.51)	8.45E-16
**HDL-C,** mg/dl	55.64 (17.05)	56.41 (16.85)	53.33 (17.43)	2.84E-10
**LDL-C,** mg/dl				
Martin-Hopkins	118.97 (35.72)	121.10 (34.57)	112.54 (38.28)	9.28E-16
Friedewald	117.17 (36.32)	119.70 (35.11)	109.53 (38.77)	3.83E-19
Sampson	119.71 (36.29)	122.14 (35.17)	112.38 (38.57)	5.84E-18
**RC,** mg/dl				
Martin-Hopkins	23.33 (8.92)	22.90 (8.69)	24.62 (9.47)	2.45E-10
Friedewald	25.13 (13.51)	24.30 (13.08)	27.63 (14.45)	1.54E-15
Sampson	22.59 (11.79)	21.86 (11.45)	24.78 (12.49)	3.90E-17
**RC to TC Ratio,** %				
Martin-Hopkins	11.15 (8.86, 14.31)	10.71 (8.61, 13.74)	12.47 (9.92, 15.51)	1.91E-28
Friedewald	11.21 (7.93, 16.17)	10.63 (7.62, 15.36)	13.27 (9.44, 18.39)	4.44E-28
Sampson	10.30 (7.39, 14.56)	9.74 (7.04, 13.83)	12.13 (8.75, 16.38)	2.45E-29
**RC to LDL-C Ratio,** %				
Martin-Hopkins	18.78 (14.95, 24.31)	17.95 (14.40, 23.27)	21.32 (16.93, 27.39)	1.49E-33
Friedewald	18.81 (13.15, 28.14)	17.65 (12.50, 26.35)	22.74 (16.06, 33.69)	3.35E-33
Sampson	17.15 (12.16, 24.86)	15.99 (11.51, 23.47)	20.70 (14.80, 29.47)	5.77E-34

*Notes*: Percentages, Mean value, and Standard deviation were weighted and accounted for the complex sampling design. Sample size was unweighted. *BMI* Body mass index RC Remnant cholesterol, *TC* Total cholesterol, *LDL-C* Low-density lipoprotein cholesterol, *HEI-2015* Healthy Eating Index-2015, *CVD* Cardiovascular disease, *DM* Diabetes mellitus, *eGFR* Estimated glomerular filtration rate, *SBP* Systolic blood pressure, *DBP* Diastolic blood pressure

**Fig. 2:**
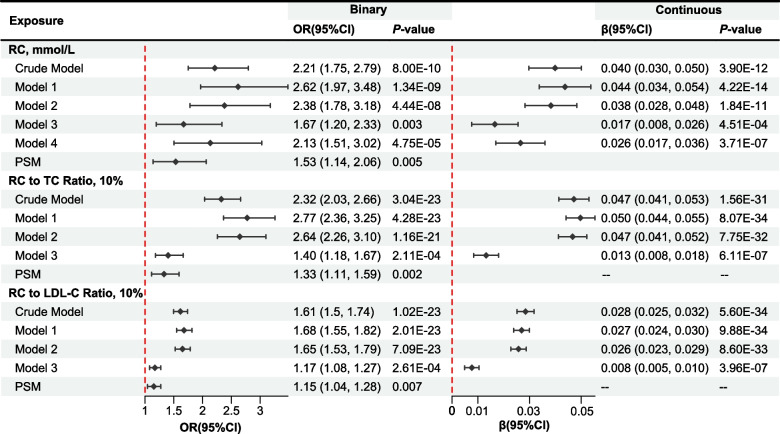
Forest Plot for Association of RC, RC-to-TC ratio, and RC-to-LDL-C ratio with the Frailty. The value of LDL-C was calculated using the Martin-Hopkins equation. Model 1 adjusted for age (continuous), gender (female and male), ethnicity (white, black, or other), education level (below high school, or high school and above), family income (poor, near poor, or non-poor), and marital status (non-married or married). Model 2 adjusted for the variables in model 1 plus smoking status (never, former, and current) and HEI-2015 (quartile). Model 3 adjusted for the variables in model 2 plus BMI (continuous), SBP (continuous), DBP (continuous), eGFR level (≥ 90, 60 to 89, and < 60 ml/min per 1.73 m2), CVD (no or yes), DM (no or yes), statins use (no or yes), anti-Diabetic drug use (no or yes), and anti-Hypertensive drug use (no or yes). Model 4 adjusted for the variables in model 3 plus TC (continuous) and LDL-C (continuous). OR, odds ratio; CI, confidence interval; RC, remnant cholesterol; TC, total cholesterol; LDL-C, low-density lipoprotein cholesterol

Upon adjusting for potential confounders, the study found a positive correlation between elevated serum RC levels and frailty as determined by FI (Fig. 2). In particular, the adjusted odds ratio (OR) of frailty for RC (calculated by the Martin-Hopkins equation) was determined as 1.67 (95% CI = 1.20 to 2.33, *P* = 0.003). A 1 mmol/L increase in serum levels of RC was associated with a 0.017 unit increase in continuous FI (β = 0.017 per 1 mmol/L increase in RC levels, 95% CI = 0.008 to 0.026, *P* = 4.51E-04). No multicollinearity was detected among the independent variables in model 3, as confirmed by the GVIF values (Supplementary Table 3). The relationship remained statistically significant after further adjusting for total cholesterol and LDL-C (model 4; Fig. 2). PSM achieved a satisfactory balance between the covariates in non-frail and frail groups (Supplementary Table 4), and RC maintained a significant correlation with the likelihood of frailty (OR = 1.53 per 1 mmol/L increase in RC levels, 95% CI = 1.14 to 2.06, *P* = 0.005). Nevertheless, there was no notable correlation detected between higher serum levels of RC and frailty as defined by FP (OR = 1.32 per 1 mmol/L increase in RC levels, 95% CI = 0.87 to 2.01, *P* = 0.192), as shown in Supplementary Table 5. Additionally, our study revealed a positive correlation between elevated levels of the RC-to-TC ratio, the RC-to-LDL-C ratio, and frailty as defined by both FI (Fig. 2) and FP (Supplementary Table 5).

**Fig. 3:**
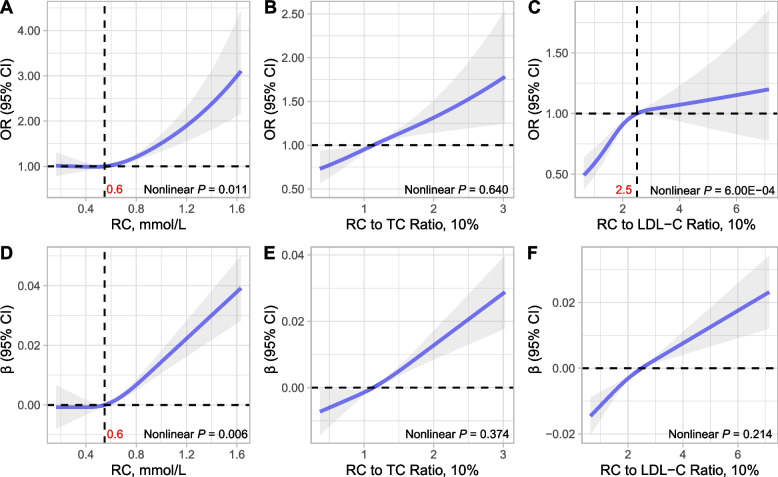
Restricted Cubic Spline Curves for Association of RC with the Frailty. (**A**) Association of RC with the likelihood of frailty, (**B**) Association of RC to TC ratio with the likelihood of frailty, (**C**) Association of RC to LDL-C ratio with the likelihood of frailty. (**D**) Association of RC with the FI, (**E**) Association of RC to TC ratio with the FI, (**F**) Association of RC to LDL-C ratio with the FI. The models adjusted for age (continuous), gender (female and male), ethnicity (white, black, or other), ethnicity (white, black, or other), education level (below high school, or high school and above), marital status (non-married or married), smoking status (never, former, and current), HEI-2015 (quartile), BMI (continuous), SBP (continuous), DBP (continuous), eGFR level (≥ 90, 60 to 89, and < 60 ml/min per 1.73 m2), CVD (no or yes), DM (no or yes), statins use (no or yes), anti-Diabetic drug use (no or yes), and anti-Hypertensive drug use (no or yes). For RC, the model additionally adjusted for TC (continuous) and LDL-C (continuous). OR, odds ratio; CI, confidence interval; RC, remnant cholesterol; TC, total cholesterol; LDL-C, low-density lipoprotein cholesterol

Figure 3 depicts the use of restricted cubic splines to illustrate the dose–response association between serum RC levels and the likelihood of frailty. The findings indicate that the dose–response relationship of serum RC levels (non-linear *P* = 0.011) and the RC-to-LDL-C ratio (non-linear *P* = 6.00E-04) with frailty displayed non-linearity. Conversely, the relationship between the RC-to-TC ratio and the likelihood of frailty was linear (non-linear *P* = 0.620). Two-segment piecewise regression models with inflection point of the curve were fitted to quantify the effect of RC above and below the inflection point. Importantly, the likelihood of frailty remained relatively constant until approximately 0.55 mmol/L of RC (OR = 1.47 per 1 mmol/L increase in RC levels, 95% CI = 0.40 to 5.43, *P* = 0.564) before observing a swift increase (OR = 2.83 per 1 mmol/L increase in RC levels, 95% CI = 1.54 to 5.20, *P* = 0.001; Supplementary Table 6). Conversely, the likelihood of frailty amplified until around 0.25 (OR = 1.38 per 1 mmol/L increase in RC levels, 95% CI = 1.17 to 1.63, *P* = 2.91E-04), after which the increase plateaued (OR = 1.06 per 1 mmol/L increase in RC levels, 95% CI = 0.90 to 1.25, *P* = 0.468), as displayed in Supplementary Table 6.

### Subgroups and sensitivity analyses

**Fig. 4:**
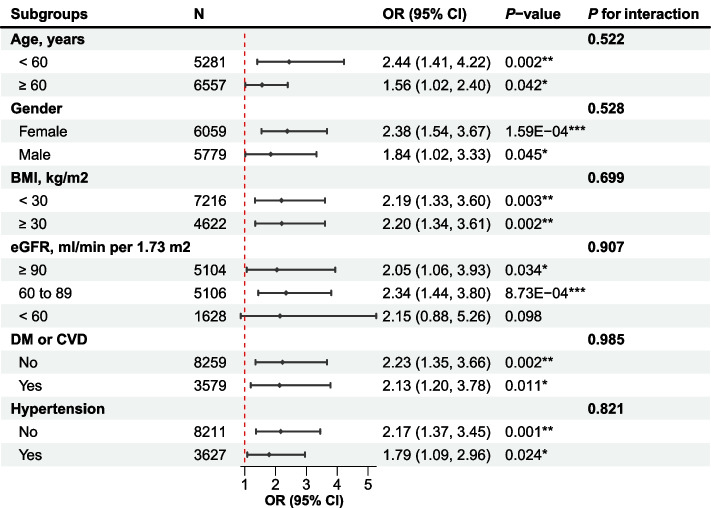
Forest Plot for Subgroup Analyses of the Association Between RC and Frailty. Adjusted for age (continuous), gender (female and male), ethnicity (white, black, or other), education level (below high school, or high school and above), marital status (non-married or married), smoking status (never, former, and current), HEI-2015 (quartile), BMI (continuous), SBP (continuous), DBP (continuous), eGFR level (≥ 90, 60 to 89, and < 60 ml/min per 1.73 m^2^), CVD (no or yes), DM (no or yes), statins use (no or yes), anti-Diabetic drug use, anti-Hypertensive drug use, TC (continuous), and LDL-C (continuous), except the subgroup variable. *P*-value for interaction was corrected for false discovery rate (FDR)-based multiple hypothesis testing. *, < 0.05; **, < 0.01; ***, < 0.001

The directionality of effect estimates across all evaluated subgroups aligned with the overall outcomes (Fig. 4 and Supplementary Fig. 2). Of significance was the association between serum levels of RC and the likelihood of frailty, which demonstrated statistical significance irrespective of age subgroup: OR of 2.44 (95% CI = 1.41 to 4.22, *P* = 0.002) for middle-aged adults (< 60 years), and 1.56 (95% CI = 1.02 to 2.40, *P* = 0.042) for older adults (≥ 60 years). Corresponding trends were observed for the RC-to-TC ratio and RC-to-LDL-C ratio (Supplementary Fig. 2). No significant interactions were detected (Fig. 4 and Supplementary Fig. 2).

Three distinct sensitivity analyses were conducted to verify the robustness of the principal findings. First, the association of RC levels with frailty, as determined by the modified FI, was verified (Supplementary Table 7). Second, missing data were addressed via two methods, direct deletion and multiple imputation, confirming that the associations of RC, RC-to-TC ratio, and RC-to-LDL-C ratio with the likelihood of frailty remained consistent irrespective of the method applied (Supplementary Table 8 and Supplementary Table 9). Lastly, the congruence between the results drawn from Friedewald and Sampson equations with the primary findings was established (Supplementary Table 10 and Supplementary Fig. 3).

### Mendelian randomization analysis for causal association of RC with frailty

A total of 51 RC-related, 16 FI-related, and 36 FP-related SNPs, all with F-statistics exceeding 10, were employed as genetic instrumental variables (Supplementary Tables 11–13). The multiplicative random-effects IVW analysis illuminated a positive correlation between the genetically inferred heightened level of RC and an increase in FI (β = 0.059 per 1 mmol/L increase in RC levels, 95% CI = 0.033 to 0.085, *P* = 1.05E-05; Table 2). However, no causal associations between RC and FP were found (Supplementary Table 14).

**Table 2:** Bidirectional MR analyses for the association between RC and FI

Methods	β (95% CI)	*P*-value
**Effect of RC on FI**		
IVW	0.059 (0.033, 0.085)	1.05E-05^***^
MR-Egger	0.058 (0.015, 0.100)	0.011^*^
Weighted median	0.063 (0.032, 0.093)	5.18E-05^***^
Simple median	0.083 (0.050, 0.117)	1.61E-06^***^
MR-PRESSO	0.059 (0.033, 0.085)	5.67E-05^***^
MRlap	0.061 (0.036, 0.086)	9.81E-07^***^
**Effect of FI on RC**		
IVW	-0.140 (-0.293, 0.014)	0.075
MR-Egger	-0.989 (-1.477, -0.502)	0.001^**^
Weighted median	-0.137 (-0.300, 0.025)	0.113
Simple median	-0.079 (-0.235, 0.077)	0.358
MR-PRESSO	-0.140 (-0.293, 0.014)	0.095
MRlap	0.030 (-0.170, 0.230)	0.772

*Notes*: The effect size (β) is per 1 mmol/L increase in RC. *IVW* Inverse-variance-weighted, *MR-PRESSO* Mendelian Randomization-Pleiotropy RESidual Sum and Outlier, *CI* Confidence interval, *RC* Remnant cholesterol, *FI* Frailty index. *, < 0.05; **, < 0.01; ***, < 0.001

The multivariable MR analysis substantiated a direct effect of RC on FI. Following adjustment for TC and LDL-C via multivariable MR, the results maintained alignment with the principal findings (β = 0.086 per 1 mmol/L increase in RC levels, 95% CI = 0.012 to 0.161, *P* = 0.024; Table 3). The associations of RC with FI remained stable, irrespective of adjustment for CHD, HF, stroke, T2DM, BMI, SBP, or DBP (Table 3).

**Table 3:** Multivariable MR analyses for the Causal Effect of RC on FI

Adjustment	β (95% CI)	*P*-value
TC and LDL-C	0.086 (0.012, 0.161)	0.024^*^
CHD	0.032 (0.006, 0.058)	0.001^**^
HF	0.043 (0.018, 0.067)	5.67E-04^***^
Stroke	0.051 (0.025, 0.076)	9.05E-05^***^
T2DM	0.068 (0.027, 0.108)	0.001^**^
BMI	0.069 (0.031, 0.107)	2.11E-04^***^
SBP	0.047 (0.021, 0.073)	4.30E-04
DBP	0.041 (0.015, 0.067)	0.002^**^

*Notes*: The effect size (β) is per 1 mmol/L increase in RC. *LDL-C* Low-density lipoprotein cholesterol, *TC* Total cholesterol, *T2DM* Type 2 diabetes mellitus, *HF* Heart failure, *BMI* Body mass index. *, < 0.05; **, < 0.01; ***, < 0.001

Furthermore, the accuracy of the inferred causal direction was verified using the MR-Steiger test for directionality. In addition, no significant causal effect of FI on RC was noted (Table 2). The MRlap analysis comparing observed and corrected effects affirmed consistency (Table 2).

High statistical heterogeneity was observed among individual SNP estimates in the analysis of FI (IVW, Cochran’s Q Statistic = 95.04, I^2^ = 47.39%, *P* = 1.27E-04; MR-Egger, Cochran’s Q Statistic = 95.03, I^2^ = 48.44%, *P* = 8.92E-05) and FP (IVW, Cochran’s Q Statistic = 88.18, I^2^ = 60.31%, *P* = 1.76E-06; MR-Egger, Cochran’s Q Statistic = 87.78, I^2^ = 61.27%, *P* = 1.22E-06). Funnel asymmetry was suggested by the visual inspection of the funnel plot (Supplementary Fig. 4). Upon removal of heterogeneity-associated SNPs (rs653178, rs9682783, rs102275, and rs6601299), heterogeneity was eliminated (IVW, Cochran’s Q Statistic = 61.26, I^2^ = 24.91%, *P* = 0.065; MR-Egger, Cochran’s Q Statistic = 0.054, I^2^ = 26.53%, *P* = 0.054), while the causal association maintained significance (Supplementary Table 15).

MR-Egger intercept test provided no evidence for directional pleiotropy in assessing the causal association of RC with FI (Egger intercept = 7.55 × 10^–5^, *P* = 0.954). Utilizing the PhenoScanner tool, three SNPs (rs12916, rs4876611, and rs653178) that had associations with potential confounders (BMI, SBP, and/or DBP) were identified in the publicly available summary-level GWAS data. An additional sensitivity analysis was conducted, excluding these SNPs and the four previously mentioned SNPs, which yielded similar results of all MR methods (Supplementary Table 16) and no significant heterogeneity was detected (IVW, Cochran’s Q Statistic = 58.05, I^2^ = 24.21%, *P* = 0.076; MR-Egger, Cochran’s Q Statistic = 57.89, I^2^ = 25.73%, *P* = 0.064; Supplementary Table 16).

## Discussion

This study determined that higher establishes a correlation between elevated RC levels and an increased susceptibility to frailty among middle-aged and older adults. Both observational and MR studies corroborate this, with sensitivity analysis further strengthening the validity of the findings. Additionally, a threshold effect was observed in the relationship between RC and frailty.

The Rookwood frailty index and the Fried frailty phenotype are the two most commonly employed instruments for identifying frailty. The results of the observational analysis indicated a consistent trend in the effects of both the FI and the FP. However, the MR analysis revealed no significant association between RC and FP. These findings align with those reported in a recent study [39]. While the converging evidence for risk factors between the two measurements is regarded as indicative of validity, the two measurements should be deemed complementary but not equivalent [40, 41]. The FP primarily focuses on physical functioning, whereas the FI encompasses accumulation of health deficits, such as coronary heart disease, angina, heart attack, stroke, and T2DM. Furthermore, the sensitivity analysis of modified FI, excluding items related to cardiometabolic disease, suggested high levels of serum RC might contribute to a high burden of multimorbidity in middle-aged and older adults.

Despite a lack of direct epidemiological evidence linking serum circulating RC levels to frailty, recent MR studies have spotlighted the influential role of elevated LDL-C levels in inducing frailty [9]. Substantial increases in RC levels have been documented in adults consuming high-fat diets [42]. The same diets administered to mice resulted in a heightened frailty level [43], while simultaneously diminishing the anti-frailty benefits of intermittent fasting [44]. Consequently, this indirect evidence suggests a connection between higher RC levels and a heightened frailty risk, which this study substantiates.

Although increased serum RC levels are regarded as a potent independent risk factor for CVD [45, 46], this analysis reveals that the association between serum RC levels and frailty persists, even after adjusting for CVD and T2DM. This suggests that the contribution of RC to frailty risk is not exclusively attributed to a higher susceptibility to CVD. Furthermore, the results from our epidemiological studies and multivariable MR confirm that this association remains significant, regardless of total cholesterol or LDL-C levels.

Individuals exhibiting high RC levels, and therefore a greater frailty risk, should be promptly identified and intervened, especially those with underlying cardiometabolic conditions such as coronary heart disease and diabetes. The findings suggest that RC is a risk factor for frailty, which should urge clinicians and researchers to prioritize attention toward such individuals. This becomes particularly vital as statin therapy, commonly used to lower LDL-C and prevent cardiovascular incidents, has minimal effect on reducing RC. As such, focusing on managing elevated RC levels is critical to counteract its potential role in accelerating aging and frailty.

## Strengths and limitations

This study offers multiple points of strength. This study is the first to investigate the correlation between RC and frailty among non-institutionalized middle-aged and older adults. While the cross-sectional design of the observational data inherently restricts causal interpretation, efforts have been undertaken to strengthen causal inferences through the robustness of MR analysis. Furthermore, this study has found an association of the proportion of RC to TC or LDL-C with frailty, underscoring a saturation effect.

Conversely, this research also exhibits certain limitations that warrant acknowledgment. The LDL-C levels reported in the observational study were not direct measurements but rather, estimated values, thereby introducing potential measurement bias. However, this risk has been mitigated by employing three different equations to predict LDL-C levels and subsequently comparing the results. Additionally, the study’s reliance on NHANES data that predominantly features individuals of white ancestry could potentially restrict the broader applicability of the findings to diverse populations. Another limitation pertains to the non-linear MR design, which was constrained by the unavailability of individual-level GWAS data that is publicly accessible. Lastly, the FI utilized in this research was dependent on self-reported data, a factor that could lead to potential reporting bias.

## Conclusion

To summarize, this research, through a combined observational and MR study, provides compelling evidence that an elevated RC level amplifies the risk of frailty in middle-aged and older adults. Interventions aimed at decreasing RC levels and the proportion of RC to TC or LDL-C could potentially confer benefits in the prevention and management of frailty. This underscores the importance of developing innovative therapies aiming at reducing the risk of frailty.

### Supplementary Information

**Additional file 1:** **Table S1.** The 49-Item Defects to Calculate Frailty Index. **Table S2.** Phenotype descriptions and distributions. **Table S3.** Generalized Variance Inflation Factor. **Table S4**. Population Characteristics After Propensity Score Matching (*N* = 5236). **Table S5.** Association of RC, RC-to-TC Ratio, and RC-to-LDL-C Ratio with the Fried Frailty Phenotype. **Table S6.** The results of the two-piecewise logistic regression model. **Table S7.** Association of RC, RC-to-TC Ratio, and RC-to-LDL-C Ratio with the Frailty as Defined by Modified Frailty Index. **Table S8.** Association of RC with frailty based on multiple imputation. **Table S9**. Association of RC with the risk of frailty based on direct deletion. **Table 10.** Association of RC with the FI (Continuous). **Table S11.** Instrumental Variables for RC. **Table S12.** Instrumental Variables for Frailty Index. **Table S13**. Instrumental Variables for Fried Frailty Phenotype. **Table S14.** Bidirectional MR Analyses for the Association between RC and Fried Frailty Phenotype. **Table S15**. MR Results for Effect of RC on FI Removing four SNPs. **Table 16**. MR Results for Effect of RC on FI Removing six SNPs. **Fig. S1.** Correlation of LDL-C Calculated by Different Equations. **Fig. S2.** Forest Plot for Subgroup Analyses. **Fig. S3.** Forest Plot for Association of RC, RC-to-TC ratio, and RC-to-LDL-C ratio with Frailty. **Fig. S4.** Funnel plot. (A) Raw; (B) Removing rs653178, rs9682783, rs102275, and rs6601299; (C) Removing rs12916, rs4876611, rs653178, rs9682783, rs102275, and rs6601299.

**Additional file 2.**

**Additional file 3.**

## Data Availability

This data can be found at: Publicly available datasets were analyzed in this study. The summary-level GWAS data of frailty index was downloaded from the NHGRI-EBI Catalog of human genome-wide association studies (GWAS Catalog, https://www.ebi.ac.uk/gwas/home), including remnant cholesterol (ID: GCST90092943), total cholesterol (ID: GCST90092985), low-density lipoprotein cholesterol (ID: GCST90092883), frailty index (ID: GCST90020053), body mass index (ID: GCST006900), CHD (ID: GCST003116), T2DM (ID: GCST006867), HF (ID: GCST009541), stroke (ID: GCST005838), SBP (ID: GCST006624), and DBP (ID: GCST006630). The NHANES data was downloaded from the National Center for Health Statistics website (https://www.cdc.gov/nchs/nhanes/index.htm).
