# Supplementary material for: Association of remnant cholesterol with frailty: findings from observational and Mendelian randomization analyses
Source: Lipids Health Dis. 2023 Aug 3;22:115. doi: 10.1186/s12944-023-01882-4 (PMC10399004; doi:10.1186/s12944-023-01882-4)
Supplement: Supplementary file 1 — Additional file 1: Table S1. The 49-Item Defects to Calculate Frailty Index. Table S2. Phenotype descriptions and distributions. Table S3. Generalized Variance Inflation Factor. Table S4. Population Characteristics After Propensity Score Matching (N = 5236). Table S5. Association of RC, RC-to-TC Ratio, and RC-to-LDL-C Ratio with the Fried Frailty Phenotype. Table S6. The results of the two-piecewise logistic regression model. Table S7. Association of RC, RC-to-TC Ratio, and RC-to-LDL-C Ratio with the Frailty as Defined by Modified Frailty Index. Table S8. Association of RC with frailty based on multiple imputation. Table S9. Association of RC with the risk of frailty based on direct deletion. Table 10. Association of RC with the FI (Continuous). Table S11. Instrumental Variables for RC. Table S12. Instrumental Variables for Frailty Index. Table S13. Instrumental Variables for Fried Frailty Phenotype. Table S14. Bidirectional MR Analyses for the Association between RC and Fried Frailty Phenotype. Table S15. MR Results for Effect of RC on FI Removing four SNPs. Table 16. MR Results for Effect of RC on FI Removing six SNPs. Fig. S1. Correlation of LDL-C Calculated by Different Equations. Fig. S2. Forest Plot for Subgroup Analyses. Fig. S3. Forest Plot for Association of RC, RC-to-TC ratio, and RC-to-LDL-C ratio with Frailty. Fig. S4. Funnel plot. (A) Raw; (B) Removing rs653178, rs9682783, rs102275, and rs6601299; (C) Removing rs12916, rs4876611, rs653178, rs9682783, rs102275, and rs6601299. [file 12944_2023_1882_MOESM1_ESM.docx]

**Supplementary Materials**

**Supplementary Table 1. The 49-Item Defects to Calculate Frailty Index**

| **Defects** | **Value or Categories** | **Score** |
| --- | --- | --- |
| **Cognition** |  |  |
| 1. Experience confusion/memory problems | No, yes | Categorized 0/1 |
| **Dependence** |  |  |
| 2. Managing money | No difficulty, difficulty | Categorized 0/1 |
| 3. Stooping, crouching, kneeling |  |  |
| 4. Lifting or carrying |  |  |
| 5. House chore |  |  |
| 6. Preparing meals |  |  |
| 7. Standing up from armless chair |  |  |
| 8. Getting in and out of bed difficulty |  |  |
| 9. Using fork, knife, drinking from cup |  |  |
| 10. Dressing yourself |  |  |
| 11. Standing for long periods difficulty |  |  |
| 12. Grasp/holding small objects |  |  |
| 13. Attending social event |  |  |
| 14. Push or pull large objects |  |  |
| 15. Walking for a quarter mile difficulty |  |  |
| 16. Walking up 10 steps difficulty |  |  |
| **Depressive Symptoms** |  |  |
| 17. Have little interest in doing things | Nearly every day, more than half the days, several days, not at all | Nearly every day = 1, More than half the days = 0.66, Several days = 0.33, Not at all = 0 |
| 18. Feeling down, depressed, or hopeless |  |  |
| 19. Trouble sleeping or sleeping too much |  |  |
| 20. Feeling tired or having little energy |  |  |
| 21. Poor appetite or overeating |  |  |
| 22. Feeling bad about yourself |  |  |
| 23. Trouble concentrating on things |  |  |
| **Comorbidities** |  |  |
| 24. Arthritis | No, suspect, yes | No = 0, Suspect = 0.5, Yes = 1 |
| 25. Thyroid problems |  |  |
| 26. Chronic bronchitis |  |  |
| 27. Cancer |  |  |
| 28. Congestive heart failure |  |  |
| 29. Coronary heart disease |  |  |
| 30. Angina |  |  |
| 31. Heart attack |  |  |
| 32. Stroke |  |  |
| 33. Blood pressure |  |  |
| 34. Diabetes |  |  |
| 35. Weak/failing kidneys |  |  |
| 36. Urinary Leakage |  |  |
| **Hospital Utilization and Access to Care** |  |  |
| 37. Self-rated health | Fair, poor, excellent, very good, good | Fair or poor = 1; excellent, very good, or good = 0 |
| 38. Health now compared with 1 year ago | Worse, About the same, better | Worse = 1; About the same, better = 0 |
| 39. Overnight hospital patient in past year | no, yes | Categorized 0/1 |
| 40. Frequency of health care use during past year | 0, 1, 2, 3, 4, 5, and more | None = 0, 1 to 5 = 0.5, More than 5 = 1 |
| 41. Number of prescribed medications |  | None = 0, 1 to 4 = 0.5, ≥5 = 1 |
| **Physical Performance and Anthropometry** |  |  |
| 42. Body mass index | Continuous values | <18.5 or ≥30 = 1, 25 to 30 = 0.5, 18.5 to 25 = 0 |
| 43. Handgrip strength |  | Male:  For BMI ≤ 24, GS ≤ 29 = 1;  For BMI 24.1 to 28, GS ≤ 30 = 1;  For BMI >28, GS ≤ 32 = 1.  Female:  For BMI ≤23, GS ≤17 = 1;  For BMI 23.1 to 26, GS ≤17.3 = 1;  For BMI 26.1 to 29, GS ≤ 18 = 1;  For BMI>29, GS ≤ 21 = 1. |
| **Laboratory Values** |  |  |
| 44. Glycohemoglobin (%) | Continuous values | 0% to 5.7% = 0, >5.7% = 1 |
| 45. Red blood cell count (million cells/ml) |  | Male: 4.7 to 6.1 = 0, Other = 1  Female: 4.2 to 5.4 = 0, Other = 1 |
| 46. Hemoglobin (g/dL) |  | Male: 13.5 to 18 = 0, Other = 1  Female: 12 to 16 = 0, Other = 1 |
| 47. Red cell distribution width (%) |  | 11.6 to 14.6 = 0, Other = 1 |
| 48. Lymphocyte percent (%) |  | 20 to 40 = 0, Other = 1 |
| 49. Segmented neutrophils percent (%) |  | 40 to 80 = 0, Other = 1 |

Notes: BMI = Body mass index; GS = grip strength.

**Supplementary Table 2**. Phenotype descriptions and distributions

| **Phenotypes** | **Study / Consortium** | **Sample Size** | **N Cases** | **N Controls** | **Population** | **PMID** |
| --- | --- | --- | --- | --- | --- | --- |
| **Lipids** |  |  |  |  |  |  |
| RC | UK BioBank | 115,082 | -- | -- | European ancestry | 35213538 |
| LDL-C |  | 115,078 | -- | -- | European ancestry | 35213538 |
| TC |  | 115,078 | -- | -- | European ancestry | 35213538 |
| **Frailty** |  |  |  |  |  |  |
| Frailty index | UK Biobank | 175,226 | -- | -- | European ancestry | 34431594 |
| Frailty phenotype |  | 386,565 | -- | -- | European ancestry | 36928559 |
| **Others** |  |  |  |  |  |  |
| T2DM | DIAGRAM, GERA, and UK Biobank | 655,666 | 61,714 | 593,952 | European ancestry | 30054458 |
| CHD | CARDIoGRAMplusC4D | 194,427 | 63,746 | 130,681 | European ancestry | 23202125 |
| BMI | UK Biobank | 681,275 | -- | -- | European ancestry | 30124842 |
| HF | HERMES | 977,323 | 47,309 | 930,014 | European ancestry | 31919418 |
| Stroke | MEGASTROKE | 446,696 | 40,585 | 406,111 | European ancestry | 29531354 |
| SBP | International Consortium of Blood Pressure | 757,601 | -- | -- | European ancestry | 30224653 |
| DBP | International Consortium of Blood Pressure | 757,601 | -- | -- | European ancestry | 30224653 |

**Notes:** RC, remnant cholesterol; LDL-C, Low Density Lipoprotein Cholesterol; TG, Triglycerides; T2DM, Type 2 diabetes; CHD, Coronary heart disease; BMI, body mass index; HF, heart failure; HERMES, Heart Failure Molecular Epidemiology for Therapeutic Targets; RC, remnant cholesterol.

**
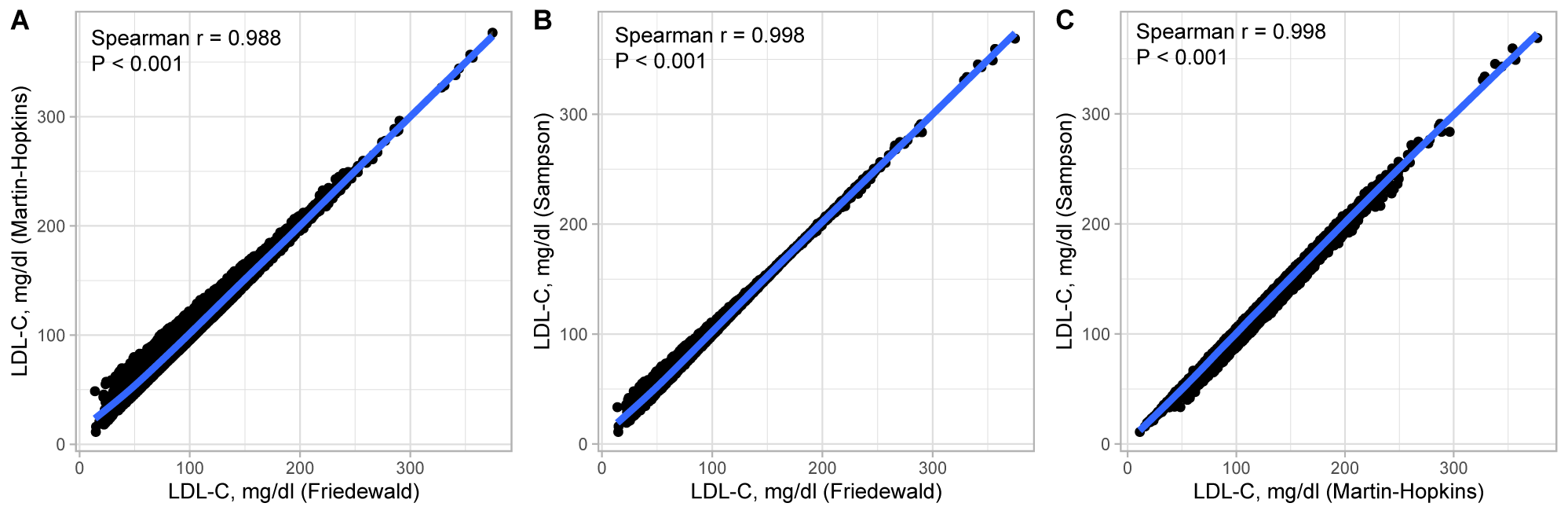
**

**Supplementary Figure 1. Correlation of LDL-C Calculated by Different Equations.**

**Supplementary Table 3. Generalized Variance Inflation Factor.**

| **Variables** | **Martin-Hopkins** | **Friedewald** | **Sampson** |
| --- | --- | --- | --- |
| RC | 1.550661 | 1.490321 | 1.495125 |
| Age | 3.096317 | 3.086574 | 3.082761 |
| Gender | 1.312285 | 1.312683 | 1.316134 |
| Ethnicity | 3.596304 | 3.530045 | 3.592478 |
| Education level | 1.894864 | 1.919901 | 1.904843 |
| Marital status | 1.449242 | 1.451853 | 1.45225 |
| Family income | 2.111297 | 2.129039 | 2.103543 |
| Smoke status | 1.934732 | 1.93292 | 1.929467 |
| HEI-2015 | 2.904568 | 2.899741 | 2.926626 |
| SBP | 2.399639 | 2.403965 | 2.397075 |
| DBP | 1.992475 | 2.004263 | 1.996371 |
| BMI | 1.461034 | 1.463611 | 1.464575 |
| eGFR | 2.482163 | 2.499631 | 2.493665 |
| T2DM | 5.523062 | 5.519801 | 5.490222 |
| CVD | 1.557383 | 1.55016 | 1.555751 |
| Statins use | 2.24569 | 2.185067 | 2.179053 |
| Anti-Diabetic drug use | 4.560349 | 4.565998 | 4.557582 |
| Anti-Hypertensive drug use | 1.47483 | 1.483692 | 1.479167 |

**Notes:** RC, remnant cholesterol; HEI-2015, Healthy Eating Index-2015; SBP, systolic blood pressure; DBP, diastolic blood pressure; BMI, body mass index; CVD, cardiovascular disease; T2DM, Type 2 diabetes mellitus; eGFR, estimated glomerular filtration rate.

**Supplementary Table 4. Population Characteristics After Propensity Score Matching (N = 5236)**

| **Characteristics** | **Total**  **(N = 5236)** | **Non-frailty**  **(N = 2618)** | **Frailty**  **(N = 2618)** | ***P*-value** | **SMD (95% CI)** |
| --- | --- | --- | --- | --- | --- |
| **Age**, years | 61.32 (12.18) | 61.40 (12.05) | 61.25 (12.32) | 0.537 | 0.01 (0.01, 0.01) |
| **Gender** |  |  |  | 0.943 | 0 (0.00, 0.00) |
| Female | 2960 (59.43%) | 1517 (59.50%) | 1443 (59.36%) |  |  |
| Male | 2276 (40.57%) | 1101 (40.50%) | 1175 (40.64%) |  |  |
| **Ethnicity** |  |  |  | 0.743 | 0.02 (0.02, 0.02) |
| White | 2426 (71.08%) | 1177 (71.42%) | 1249 (70.74%) |  |  |
| Black | 1190 (12.29%) | 598 (11.95%) | 592 (12.63%) |  |  |
| Other | 1620 (16.63%) | 843 (16.63%) | 777 (16.63%) |  |  |
| **Education level** |  |  |  | 0.647 | 0.02 (0.02, 0.02) |
| Below High School | 1830 (24.17%) | 952 (24.51%) | 878 (23.83%) |  |  |
| High School and above | 3406 (75.83%) | 1666 (75.49%) | 1740 (76.17%) |  |  |
| **Marital status** |  |  |  | 0.603 | 0.02 (0.02, 0.02) |
| Non-Married | 2638 (46.21%) | 1329 (46.76%) | 1309 (45.66%) |  |  |
| Married | 2598 (53.79%) | 1289 (53.24%) | 1309 (54.34%) |  |  |
| **Family income** |  |  |  | 0.829 | 0.03 (0.02, 0.03) |
| Poor | 1204 (16.14%) | 627 (15.75%) | 577 (16.52%) |  |  |
| Near poor | 1824 (27.95%) | 948 (28.38%) | 876 (27.53%) |  |  |
| Non-poor | 2208 (55.91%) | 1043 (55.87%) | 1165 (55.95%) |  |  |
| **HEI-2015** |  |  |  | 0.926 | 0.03 (0.03, 0.03) |
| Quartile 1 | 1473 (29.81%) | 733 (30.17%) | 740 (29.45%) |  |  |
| Quartile 2 | 1420 (27.32%) | 728 (27.57%) | 692 (27.06%) |  |  |
| Quartile 3 | 1295 (24.37%) | 665 (23.86%) | 630 (24.88%) |  |  |
| Quartile 4 | 1048 (18.50%) | 492(18.40%) | 556 (18.60%) |  |  |
| **Smoking status** |  |  |  | 0.961 | 0.01 (0.01, 0.01) |
| Never | 2379 (42.96%) | 1172 (42.74%) | 1207 (43.17%) |  |  |
| Former | 1710 (33.10%) | 847 (33.34%) | 863 (32.85%) |  |  |
| Current | 1147 (23.95%) | 599 (23.91%) | 548 (23.98%) |  |  |
| **SBP**, mmHg | 128.40 (19.76) | 129 (19.97) | 128 (19.53) | 0.270 | 0.05 (0.05, 0.05) |
| **DBP**, mmHg | 69.69 (12.54) | 70 (12.30) | 69 (12.77) | 0.143 | 0.06 (0.06, 0.06) |
| **BMI,** kg/m2 | 30.48 (7.10) | 30 (6.94) | 31 (7.25) | 0.686 | -0.02 (-0.02, -0.02) |
| **eGFR,** ml/min per 1.73 m^2^ |  |  |  | 0.143 | 0.06 (0.06, 0.06) |
| ≥ 90 | 2077 (40.97%) | 1087 (41.28%) | 990 (40.67%) |  |  |
| 60 to 89 | 2216 (43.27%) | 1074 (43.13%) | 1,142 (43.42%) |  |  |
| < 60 | 943 (15.76%) | 457 (15.60%) | 486 (15.91%) |  |  |
| **Type 2 DM** | 1280 (21.95%) | 621 (21.59%) | 659 (22.30%) | 0.676 | 0.02 (0.02, 0.02) |
| **CVD** | 1225 (23.00%) | 550 (22.40%) | 675 (23.59%) | 0.516 | 0.03 (0.03, 0.03) |
| **Statins use** | 1818 (36.18%) | 901 (35.80%) | 917 (36.56%) | 0.705 | 0.02 (0.01, 0.02) |
| **Anti-Diabetic drug use** | 1159 (20.14%) | 555 (19.88%) | 604 (20.39%) | 0.763 | 0.01 (0.01, 0.01) |
| **Anti-Hypertensive drug use** | 579 (10.44%) | 288 (10.42%) | 291 (10.46%) | 0.972 | 0 (0.00, 0.00) |

**Notes:** PSM was employed to adjust for age (continuous), gender (female and male), ethnicity (white, black, and other), education level (below high school, high school and above), marital status (non-married or married), smoking status (never, former and current), HEI-2015 (quartile), BMI (continuous), eGFR level (≥ 90, 60 to 89, and < 60 ml/min per 1.73 m^2^), hypertension (no or yes), CVD (no or yes), DM (no or yes), statins use (no or yes), anti-Diabetic drug use (no or yes), and anti-Hypertensive drug use (no or yes). SMD, Standardized Mean Difference; CI, Confidence Interval; BMI, body mass index; HEI-2015, Healthy Eating Index-2015; CVD, cardiovascular disease; DM, diabetes mellitus; eGFR, estimated glomerular filtration rate.

**Supplementary Table 5. Association of RC, RC-to-TC Ratio, and RC-to-LDL-C Ratio with the Fried Frailty Phenotype**

| **Exposure** | **OR (95% CI)** | ***P*-value** |
| --- | --- | --- |
| RC (Martin-Hopkins), mmol/L |  |  |
| Crude Model | 1.56 (1.11, 2.20) | 0.012^*^ |
| Model 1 | 1.64 (1.10, 2.42) | 0.016^*^ |
| Model 2 | 1.49 (1.00, 2.22) | 0.051 |
| Model 3 | 1.32 (0.87, 2.01) | 0.192 |
| RC-to-TC ratio (Martin-Hopkins), 10% |  |  |
| Crude Model | 1.46 (1.23, 1.74) | 3.59E-05^***^ |
| Model 1 | 1.57 (1.29, 1.92) | 1.39E-05^***^ |
| Model 2 | 1.49 (1.22, 1.81) | 1.47E-04^***^ |
| Model 3 | 1.29 (1.03, 1.62) | 0.030^*^ |
| RC-to-LDL-C (Martin-Hopkins), 10% |  |  |
| Crude Model | 1.25 (1.16, 1.35) | 5.54E-08^***^ |
| Model 1 | 1.24 (1.14, 1.35) | 1.06E-06^***^ |
| Model 2 | 1.22 (1.12, 1.32) | 1.11E-05^***^ |
| Model 3 | 1.14 (1.03, 1.25) | 0.012^*^ |

**Notes:** Model 1 adjusted for age (continuous), gender (female and male), ethnicity (white, black, or other), education level (below high school, or high school and above), family income (poor, near poor, or non-poor), and marital status (non-married or married). Model 2 adjusted for the variables in model 1 plus smoking status (never, former, and current) and HEI-2015 (quartile). Model 3 adjusted for the variables in model 2 plus BMI (continuous), SBP (continuous), DBP (continuous), eGFR level (≥ 90, 60 to 89, and < 60 ml/min per 1.73 m^2^), CVD (no or yes), DM (no or yes), statins use (no or yes), anti-Diabetic drug use (no or yes), and anti-Hypertensive drug use (no or yes).

**Supplementary Table 6. The results of the two-piecewise logistic regression model**

| **Exposure** | **Binary** | | **Continuous** | |
| --- | --- | --- | --- | --- |
|  | **OR (95% CI)** | ***P*-value** | **β (95% CI)** | ***P*-value** |
| **RC** |  |  |  |  |
| < 0.55 mmol/L | 1.47 (0.40, 5.43) | 0.564 | 0.023 (-0.023, 0.068) | 0.329 |
| ≥ 0.55 mmol/L | 2.83 (1.54, 5.20) | 0.001 | 0.036 (0.021, 0.052) | 1.37E-05 |
| **RC to LDL-C Ratio** |  |  |  |  |
| < 0.25 | 1.38 (1.17, 1.63) | 2.91E-04 | -- | -- |
| ≥ 0.25 | 1.06 (0.90, 1.25) | 0.468 | -- | -- |


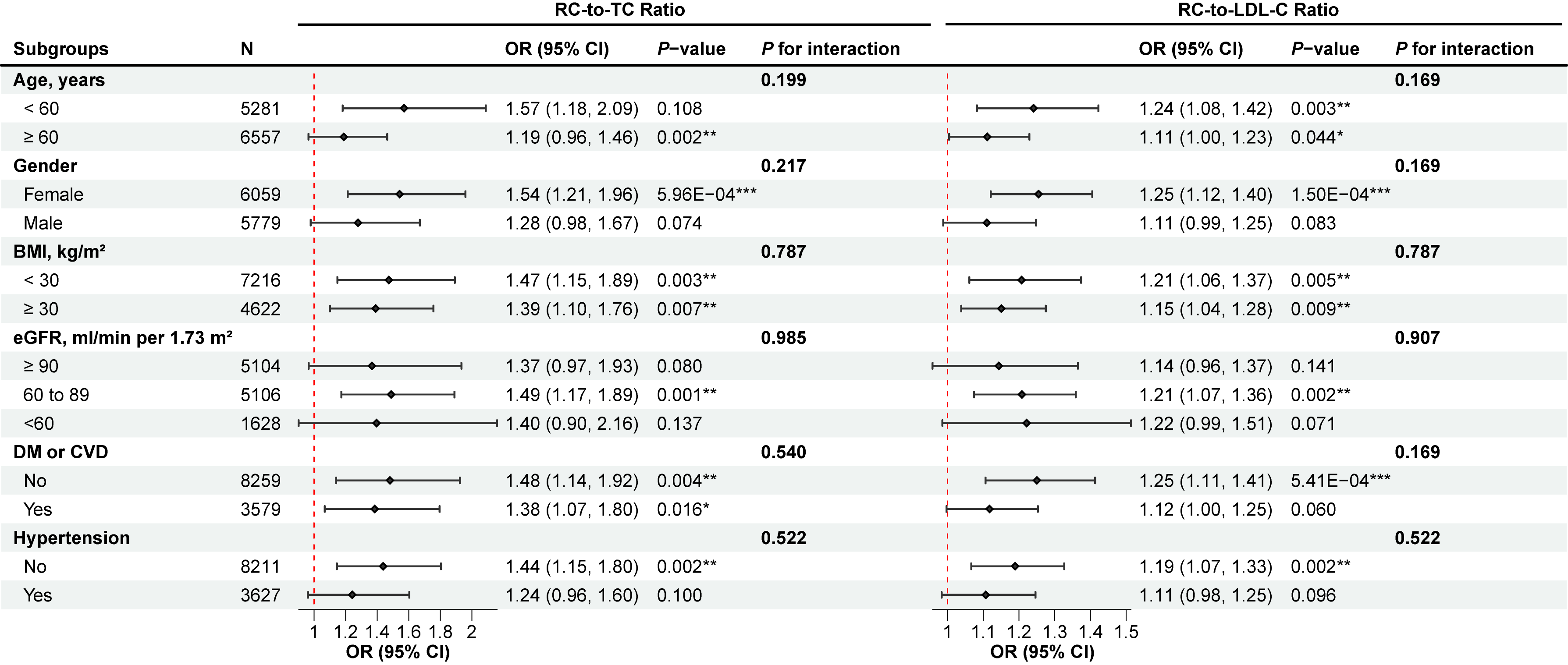


**Supplementary Figure 2.** **Forest Plot for Subgroup Analyses.** Adjusted for age (continuous), gender (female and male), ethnicity (white, black, and other), education level (below high school, high school and above), marital status (non-married or married), smoking status (never, former and current), HEI-2015 (quartile), BMI (continuous), SBP (continuous), DBP (continuous), eGFR level (≥ 90, 60 to 89, and < 60 ml/min per 1.73 m^2^), CVD (no and yes), DM (no and yes), statins use (no or yes), anti-Diabetic drug use (no or yes), and anti-Hypertensive drug use, except the subgroup variable. *, <0.05; **, <0.01; ***, <0.001.

**Supplementary Table 7. Association of RC, RC-to-TC Ratio, and RC-to-LDL-C Ratio with the Frailty as Defined by** **Modified Frailty Index**

| **Exposure** | **Binary** | | **Continuous** | |
| --- | --- | --- | --- | --- |
|  | **OR (95% CI)** | ***P*-value** | **β (95% CI)** | ***P*-value** |
| RC (Martin-Hopkins), mmol/L |  |  |  |  |
| Crude Model | 1.99 (1.63, 2.43) | 5.55E-10 | 0.041 (0.03, 0.052) | 9.53E-12 |
| Model 1 | 2.33 (1.84, 2.96) | 1.79E-10 | 0.046 (0.035, 0.056) | 1.35E-13 |
| Model 2 | 2.12 (1.66, 2.69) | 1.66E-08 | 0.040 (0.029, 0.051) | 7.10E-11 |
| Model 3 | 1.46 (1.10, 1.92) | 0.009 | 0.020 (0.009, 0.03) | 4.08E-04 |
| Model 4 | 1.91 (1.46, 2.50) | 7.87E-06 | 0.031 (0.02, 0.042) | 3.50E-07 |
| RC-to-TC ratio (Martin-Hopkins), 10% |  |  |  |  |
| Crude Model | 2.02 (1.84, 2.23) | 1.33E-27 | 0.042 (0.036, 0.048) | 1.07E-26 |
| Model 1 | 2.42 (2.16, 2.72) | 5.21E-29 | 0.047 (0.041, 0.053) | 1.16E-30 |
| Model 2 | 2.30 (2.06, 2.58) | 5.11E-27 | 0.043 (0.038, 0.049) | 1.83E-28 |
| Model 3 | 1.38 (1.20, 1.58) | 1.28E-05 | 0.015 (0.01, 0.021) | 5.14E-07 |
| RC-to-LDL-C (Martin-Hopkins), 10% |  |  |  |  |
| Crude Model | 1.50 (1.41, 1.59) | 4.41E-25 | 0.025 (0.022, 0.029) | 3.31E-28 |
| Model 1 | 1.57 (1.47, 1.67) | 2.19E-25 | 0.025 (0.022, 0.028) | 1.77E-29 |
| Model 2 | 1.54 (1.45, 1.64) | 1.03E-24 | 0.024 (0.02, 0.027) | 1.55E-28 |
| Model 3 | 1.18 (1.10, 1.28) | 1.84E-05 | 0.009 (0.006, 0.012) | 2.45E-07 |
| RC (Friedewald), mmol/L |  |  |  |  |
| Crude Model | 1.78 (1.57, 2.01) | 2.28E-15 | 0.034 (0.027, 0.041) | 3.13E-17 |
| Model 1 | 2.02 (1.74, 2.34) | 1.85E-15 | 0.037 (0.03, 0.044) | 3.17E-19 |
| Model 2 | 1.90 (1.64, 2.21) | 2.19E-13 | 0.033 (0.027, 0.04) | 2.01E-16 |
| Model 3 | 1.35 (1.14, 1.60) | 0.001 | 0.015 (0.008, 0.021) | 1.77E-05 |
| Model 4 | 1.62 (1.37, 1.91) | 1.16E-07 | 0.016 (0.01, 0.023) | 3.99E-06 |
| RC-to-TC (Friedewald), 10% |  |  |  |  |
| Crude Model | 1.50 (1.42, 1.59) | 5.11E-27 | 0.024 (0.021, 0.028) | 9.43E-26 |
| Model 1 | 1.65 (1.54, 1.76) | 1.10E-27 | 0.026 (0.023, 0.03) | 2.11E-29 |
| Model 2 | 1.61 (1.50, 1.72) | 7.79E-26 | 0.025 (0.021, 0.028) | 1.82E-27 |
| Model 3 | 1.20 (1.11, 1.31) | 1.80E-05 | 0.008 (0.005, 0.012) | 4.93E-07 |
| RC-to-LDL-C (Friedewald), 10% |  |  |  |  |
| Crude Model | 1.18 (1.14, 1.21) | 4.70E-20 | 0.009 (0.007, 0.011) | 6.82E-16 |
| Model 1 | 1.20 (1.17, 1.24) | 1.26E-20 | 0.009 (0.008, 0.011) | 5.23E-19 |
| Model 2 | 1.19 (1.16, 1.23) | 1.20E-19 | 0.009 (0.007, 0.011) | 5.42E-18 |
| Model 3 | 1.07 (1.04, 1.11) | 2.21E-05 | 0.003 (0.002, 0.004) | 9.50E-07 |
| RC (Sampson), mmol/L |  |  |  |  |
| Crude Model | 1.94 (1.68, 2.24) | 3.28E-15 | 0.040 (0.032, 0.047) | 4.15E-17 |
| Model 1 | 2.25 (1.90, 2.67) | 1.27E-15 | 0.043 (0.036, 0.051) | 1.78E-19 |
| Model 2 | 2.10 (1.77, 2.50) | 1.73E-13 | 0.039 (0.031, 0.047) | 1.47E-16 |
| Model 3 | 1.44 (1.18, 1.76) | 0.001 | 0.018 (0.010, 0.025) | 1.21E-05 |
| Model 4 | 1.80 (1.49, 2.18) | 3.41E-08 | 0.020 (0.013, 0.028) | 8.58E-07 |
| RC-to-TC (Sampson), 10% |  |  |  |  |
| Crude Model | 1.66 (1.55, 1.78) | 1.94E-28 | 0.030 (0.026, 0.035) | 1.89E-27 |
| Model 1 | 1.88 (1.74, 2.04) | 1.28E-29 | 0.033 (0.029, 0.037) | 1.92E-31 |
| Model 2 | 1.82 (1.68, 1.97) | 9.04E-28 | 0.031 (0.027, 0.035) | 1.84E-29 |
| Model 3 | 1.28 (1.16, 1.41) | 2.88E-06 | 0.011 (0.007, 0.015) | 7.29E-08 |
| RC-to-LDL-C (Sampson), 10% |  |  |  |  |
| Crude Model | 1.30 (1.25, 1.35) | 6.96E-26 | 0.016 (0.013, 0.018) | 6.91E-26 |
| Model 1 | 1.34 (1.29, 1.40) | 7.74E-27 | 0.016 (0.014, 0.018) | 8.30E-29 |
| Model 2 | 1.33 (1.28, 1.39) | 9.24E-26 | 0.015 (0.013, 0.017) | 9.88E-28 |
| Model 3 | 1.13 (1.07, 1.18) | 2.99E-06 | 0.006 (0.004, 0.007) | 4.44E-08 |

**Notes:** Model 1 adjusted for age (continuous), gender (female and male), ethnicity (white, black, or other), education level (below high school, or high school and above), family income (poor, near poor, or non-poor), and marital status (non-married or married). Model 2 adjusted for the variables in model 1 plus smoking status (never, former, and current) and HEI-2015 (quartile). Model 3 adjusted for the variables in model 2 plus BMI (continuous), SBP (continuous), DBP (continuous), eGFR level (≥ 90, 60 to 89, and < 60 ml/min per 1.73 m^2^), CVD (no or yes), DM (no or yes), statins use (no or yes), anti-Diabetic drug use (no or yes), and anti-Hypertensive drug use (no or yes). Model 4 adjusted for the variables in model 3 plus TC (continuous) and LDL-C (continuous). OR, odds ratio; CI, confidence interval; RC, remnant cholesterol; TC, total cholesterol; LDL-C, low-density lipoprotein cholesterol.

**Supplementary Table 8. Association of RC with frailty based on multiple imputation**

| **Exposure** | **Binary** | | **Continuous** | |
| --- | --- | --- | --- | --- |
|  | **OR (95% CI)** | ***P*-value** | **β (95% CI)** | ***P*-value** |
| **RC** (Martin-Hopkins), mmol/L |  |  |  |  |
| Crude Model | 2.21 (1.75, 2.79) | 2.68E-11 | 0.040 (0.030, 0.050) | 1.39E-14 |
| Model 1 | 2.62 (1.97, 3.48) | 4.19E-11 | 0.044 (0.034, 0.054) | 6.80E-18 |
| Model 2 | 2.40 (1.80, 3.21) | 3.61E-09 | 0.039 (0.029, 0.049) | 5.50E-14 |
| Model 3 | 1.74 (1.24, 2.44) | 0.001 | 0.018 (0.009, 0.027) | 9.39E-05 |
| Model 4 | 2.23 (1.57, 3.18) | 7.90E-06 | 0.028 (0.019, 0.038) | 1.01E-08 |
| **RC** (Friedewald), mmol/L |  |  |  |  |
| Crude Model | 1.95 (1.67, 2.27) | 9.22E-18 | 0.035 (0.028, 0.042) | 2.07E-25 |
| Model 1 | 2.22 (1.84, 2.67) | 1.13E-16 | 0.037 (0.031, 0.044) | 1.52E-29 |
| Model 2 | 2.10 (1.74, 2.54) | 1.84E-14 | 0.034 (0.027, 0.040) | 1.38E-24 |
| Model 3 | 1.48 (1.20, 1.83) | 3.04E-04 | 0.013 (0.008, 0.019) | 1.51E-06 |
| Model 4 | 1.56 (1.24, 1.97) | 1.41E-04 | 0.015 (0.010, 0.021) | 2.09E-07 |
| **RC** (Sampson), mmol/L |  |  |  |  |
| Crude Model | 2.15 (1.81, 2.55) | 2.74E-18 | 0.040 (0.033, 0.048) | 1.20E-25 |
| Model 1 | 2.51 (2.03, 3.10) | 1.62E-17 | 0.043 (0.036, 0.050) | 7.50E-31 |
| Model 2 | 2.36 (1.91, 2.93) | 3.95E-15 | 0.039 (0.032, 0.047) | 1.53E-25 |
| Model 3 | 1.62 (1.26, 2.08) | 1.47E-04 | 0.016 (0.010, 0.023) | 9.62E-07 |
| Model 4 | 1.76 (1.35, 2.30) | 3.21E-05 | 0.019 (0.012, 0.026) | 2.57E-08 |
| **RC** **to** **TC** **Ratio** (Martin-Hopkins), 10% |  |  |  |  |
| Crude Model | 2.32 (2.03, 2.66) | 6.02E-35 | 0.047 (0.041, 0.053) | 1.15E-56 |
| Model 1 | 2.77 (2.36, 3.25) | 1.10E-35 | 0.050 (0.044, 0.055) | 3.72E-67 |
| Model 2 | 2.65 (2.25, 3.11) | 1.12E-32 | 0.047 (0.041, 0.052) | 1.83E-61 |
| Model 3 | 1.43 (1.21, 1.70) | 4.55E-05 | 0.014 (0.009, 0.019) | 1.54E-08 |
| **RC** **to** **TC** **Ratio** (Friedewald), 10% |  |  |  |  |
| Crude Model | 1.61 (1.48, 1.74) | 8.74E-31 | 0.027 (0.024, 0.031) | 4.00E-51 |
| Model 1 | 1.76 (1.60, 1.93) | 2.58E-31 | 0.028 (0.025, 0.032) | 3.66E-60 |
| Model 2 | 1.71 (1.56, 1.88) | 2.21E-28 | 0.026 (0.023, 0.030) | 5.64E-55 |
| Model 3 | 1.22 (1.11, 1.34) | 6.72E-05 | 0.008 (0.005, 0.011) | 1.32E-08 |
| **RC** **to** **TC** **Ratio** (Sampson), 10% |  |  |  |  |
| Crude Model | 1.82 (1.65, 2.00) | 1.30E-34 | 0.034 (0.030, 0.038) | 2.91E-58 |
| Model 1 | 2.05 (1.83, 2.30) | 2.16E-35 | 0.035 (0.031, 0.039) | 1.56E-69 |
| Model 2 | 1.99 (1.77, 2.23) | 2.24E-32 | 0.033 (0.029, 0.037) | 6.44E-64 |
| Model 3 | 1.31 (1.16, 1.47) | 1.72E-05 | 0.010 (0.007, 0.014) | 8.87E-10 |
| **RC to LDL-C** **Ratio** (Martin-Hopkins), 10% |  |  |  |  |
| Crude Model | 1.61 (1.50, 1.74) | 5.14E-36 | 0.028 (0.025, 0.032) | 1.92E-64 |
| Model 1 | 1.68 (1.55, 1.82) | 1.89E-36 | 0.027 (0.024, 0.030) | 7.71E-67 |
| Model 2 | 1.65 (1.52, 1.79) | 3.53E-35 | 0.026 (0.023, 0.029) | 1.15E-64 |
| Model 3 | 1.18 (1.09, 1.28) | 8.61E-05 | 0.008 (0.005, 0.011) | 2.25E-08 |
| **RC to LDL-C Ratio** (Friedewald), 10% |  |  |  |  |
| Crude Model | 1.20 (1.14, 1.25) | 3.67E-13 | 0.010 (0.008, 0.013) | 1.73E-21 |
| Model 1 | 1.21 (1.15, 1.28) | 4.54E-13 | 0.010 (0.008, 0.012) | 3.75E-26 |
| Model 2 | 1.21 (1.14, 1.27) | 9.24E-12 | 0.010 (0.008, 0.012) | 1.04E-23 |
| Model 3 | 1.06 (1.02, 1.10) | 0.001 | 0.003 (0.002, 0.004) | 1.50E-07 |
| **RC to LDL-C Ratio** (Sampson), 10% |  |  |  |  |
| Crude Model | 1.34 (1.28, 1.42) | 2.03E-28 | 0.018 (0.015, 0.020) | 1.79E-51 |
| Model 1 | 1.39 (1.31, 1.47) | 1.87E-29 | 0.017 (0.015, 0.019) | 6.09E-59 |
| Model 2 | 1.37 (1.30, 1.46) | 5.93E-27 | 0.017 (0.014, 0.019) | 3.60E-55 |
| Model 3 | 1.12 (1.06, 1.18) | 5.19E-05 | 0.005 (0.003, 0.007) | 1.90E-09 |

**Notes:** Model 1 adjusted for age (continuous), gender (female and male), ethnicity (white, black, or other), education level (below high school, or high school and above), family income (poor, near poor, or non-poor), and marital status (non-married or married). Model 2 adjusted for the variables in model 1 plus smoking status (never, former, and current) and HEI-2015 (quartile). Model 3 adjusted for the variables in model 2 plus BMI (continuous), SBP (continuous), DBP (continuous), eGFR level (≥ 90, 60 to 89, and < 60 ml/min per 1.73 m^2^), CVD (no or yes), DM (no or yes), statins use (no or yes), anti-Diabetic drug use (no or yes), and anti-Hypertensive drug use (no or yes). Model 4 adjusted for the variables in model 3 plus TC (continuous) and LDL-C (continuous). OR, odds ratio; CI, confidence interval; RC, remnant cholesterol; TC, total cholesterol; LDL-C, low-density lipoprotein cholesterol.

**Supplementary Table 9. Association of RC with the risk of frailty based on direct deletion**

| **Exposure** | **Binary** | | **Continuous** | |
| --- | --- | --- | --- | --- |
|  | **OR (95% CI)** | ***P*-value** | **β (95% CI)** | ***P*-value** |
| **RC** (Martin-Hopkins), mmol/L |  |  |  |  |
| Crude Model | 2.27 (1.75, 2.95) | 1.05E-08 | 0.039 (0.028, 0.050) | 2.55E-10 |
| Model 1 | 2.63 (1.92, 3.59) | 1.62E-08 | 0.042 (0.031, 0.053) | 1.06E-11 |
| Model 2 | 2.40 (1.75, 3.31) | 3.95E-07 | 0.037 (0.026, 0.048) | 2.11E-09 |
| Model 3 | 1.71 (1.18, 2.49) | 0.006 | 0.017 (0.006, 0.027) | 0.002 |
| Model 4 |  |  |  |  |
| **RC** (Friedewald), mmol/L |  |  |  |  |
| Crude Model | 1.98 (1.67, 2.35) | 2.59E-12 | 0.034 (0.027, 0.041) | 6.73E-16 |
| Model 1 | 2.23 (1.81, 2.74) | 8.56E-12 | 0.036 (0.029, 0.043) | 3.06E-17 |
| Model 2 | 2.11 (1.71, 2.60) | 2.31E-10 | 0.033 (0.025, 0.040) | 1.05E-14 |
| Model 3 | 1.47 (1.17, 1.86) | 0.002 | 0.012 (0.006, 0.019) | 1.52E-04 |
| Model 4 |  |  |  |  |
| **RC** (Sampson), mmol/L |  |  |  |  |
| Crude Model | 2.19 (1.80, 2.65) | 1.26E-12 | 0.039 (0.031, 0.047) | 7.59E-16 |
| Model 1 | 2.52 (2.00, 3.18) | 2.52E-12 | 0.042 (0.034, 0.050) | 1.18E-17 |
| Model 2 | 2.37 (1.87, 3.00) | 8.70E-11 | 0.038 (0.030, 0.046) | 5.30E-15 |
| Model 3 | 1.61 (1.22, 2.13) | 0.001 | 0.015 (0.008, 0.023) | 1.17E-04 |
| Model 4 |  |  |  |  |
| **RC** **to** **TC** **Ratio** (Martin-Hopkins), 10% | 2.33 (2.00, 2.72) | 1.70E-19 | 0.044 (0.038, 0.051) | 8.25E-27 |
| Crude Model | 2.77 (2.32, 3.32) | 6.26E-20 | 0.047 (0.041, 0.054) | 2.81E-29 |
| Model 1 | 2.63 (2.20, 3.16) | 3.06E-18 | 0.044 (0.038, 0.051) | 7.01E-27 |
| Model 2 | 1.44 (1.19, 1.76) | 3.84E-04 | 0.013 (0.007, 0.018) | 1.27E-05 |
| Model 3 |  |  |  |  |
| **RC** **to** **TC** **Ratio** (Friedewald), 10% | 1.61 (1.47, 1.76) | 4.90E-18 | 0.026 (0.022, 0.029) | 1.14E-25 |
| Crude Model | 1.76 (1.58, 1.95) | 2.08E-18 | 0.027 (0.023, 0.031) | 7.54E-28 |
| Model 1 | 1.71 (1.54, 1.90) | 9.11E-17 | 0.025 (0.022, 0.029) | 1.57E-25 |
| Model 2 | 1.22 (1.10, 1.37) | 4.09E-04 | 0.007 (0.004, 0.010) | 8.66E-06 |
| Model 3 |  |  |  |  |
| **RC** **to** **TC** **Ratio** (Sampson), 10% | 1.82 (1.64, 2.03) | 1.11E-19 | 0.032 (0.028, 0.036) | 9.59E-28 |
| Crude Model | 2.05 (1.81, 2.33) | 3.79E-20 | 0.034 (0.030, 0.038) | 2.55E-30 |
| Model 1 | 1.98 (1.75, 2.25) | 1.76E-18 | 0.032 (0.028, 0.036) | 6.39E-28 |
| Model 2 | 1.31 (1.15, 1.51) | 1.54E-04 | 0.010 (0.006, 0.013) | 1.81E-06 |
| Model 3 |  |  |  |  |
| **RC to LDL-C** **Ratio** (Martin-Hopkins), 10% | 1.62 (1.50, 1.76) | 9.32E-22 | 0.027 (0.023, 0.031) | 1.46E-29 |
| Crude Model | 1.69 (1.55, 1.85) | 2.87E-21 | 0.026 (0.023, 0.029) | 6.53E-30 |
| Model 1 | 1.66 (1.52, 1.81) | 3.94E-20 | 0.025 (0.022, 0.028) | 1.71E-28 |
| Model 2 | 1.20 (1.10, 1.31) | 1.02E-04 | 0.008 (0.005, 0.011) | 1.53E-06 |
| Model 3 |  |  |  |  |
| **RC to LDL-C Ratio** (Friedewald), 10% | 1.19 (1.13, 1.26) | 2.50E-09 | 0.010 (0.008, 0.012) | 4.15E-14 |
| Crude Model | 1.21 (1.14, 1.29) | 2.63E-09 | 0.010 (0.008, 0.012) | 3.40E-16 |
| Model 1 | 1.20 (1.13, 1.28) | 1.81E-08 | 0.009 (0.007, 0.011) | 9.11E-15 |
| Model 2 | 1.07 (1.02, 1.11) | 0.002 | 0.003 (0.002, 0.004) | 8.29E-06 |
| Model 3 |  |  |  |  |
| **RC to LDL-C Ratio** (Sampson), 10% | 1.35 (1.27, 1.43) | 1.15E-17 | 0.017 (0.014, 0.019) | 1.19E-25 |
| Crude Model | 1.39 (1.31, 1.48) | 6.66E-18 | 0.017 (0.014, 0.019) | 1.48E-27 |
| Model 1 | 1.38 (1.29, 1.47) | 1.69E-16 | 0.016 (0.014, 0.018) | 9.15E-26 |
| Model 2 | 1.13 (1.06, 1.20) | 1.04E-04 | 0.005 (0.003, 0.007) | 4.07E-07 |
| Model 3 | 2.27 (1.75, 2.95) | 1.05E-08 | 0.039 (0.028, 0.050) | 2.55E-10 |

**Notes:** Model 1 adjusted for age (continuous), gender (female and male), ethnicity (white, black, or other), education level (below high school, or high school and above), family income (poor, near poor, or non-poor), and marital status (non-married or married). Model 2 adjusted for the variables in model 1 plus smoking status (never, former, and current) and HEI-2015 (quartile). Model 3 adjusted for the variables in model 2 plus BMI (continuous), SBP (continuous), DBP (continuous), eGFR level (≥ 90, 60 to 89, and < 60 ml/min per 1.73 m^2^), CVD (no or yes), DM (no or yes), statins use (no or yes), anti-Diabetic drug use (no or yes), and anti-Hypertensive drug use (no or yes). Model 4 adjusted for the variables in model 3 plus TC (continuous) and LDL-C (continuous). OR, odds ratio; CI, confidence interval; RC, remnant cholesterol; TC, total cholesterol; LDL-C, low-density lipoprotein cholesterol.

**Supplementary Table 10. Association of RC with the FI (Continuous)**

| **Exposure** | **β (95%CI)** | ***P*-value** |
| --- | --- | --- |
| **RC**, mmol/L (Friedewald) |  |  |
| Crude Model | 0.035 (0.028, 0.042) | 1.33E-18 |
| Model 1 | 0.037 (0.031, 0.044) | 2.43E-20 |
| Model 2 | 0.034 (0.027, 0.040) | 1.03E-17 |
| Model 3 | 0.013 (0.007, 0.018) | 1.56E-05 |
| Model 4 | 0.014 (0.008, 0.020) | 7.00E-06 |
| **RC**, mmol/L (Sampson) |  |  |
| Crude Model | 0.040 (0.033, 0.048) | 9.98E-19 |
| Model 1 | 0.043 (0.036, 0.050) | 5.95E-21 |
| Model 2 | 0.039 (0.032, 0.046) | 3.48E-18 |
| Model 3 | 0.015 (0.009, 0.022) | 1.12E-05 |
| Model 4 | 0.018 (0.011, 0.024) | 1.43E-06 |
| **RC to TC Ratio**, 10% (Friedewald) |  |  |
| Crude Model | 0.027 (0.024, 0.031) | 1.20E-29 |
| Model 1 | 0.028 (0.025, 0.032) | 8.40E-32 |
| Model 2 | 0.026 (0.023, 0.030) | 5.70E-30 |
| Model 3 | 0.007 (0.005, 0.010) | 4.71E-07 |
| **RC to TC Ratio**, 10% (Sampson) |  |  |
| Crude Model | 0.034 (0.030, 0.038) | 4.73E-32 |
| Model 1 | 0.035 (0.031, 0.039) | 1.80E-34 |
| Model 2 | 0.033 (0.029, 0.037) | 1.29E-32 |
| Model 3 | 0.010 (0.007, 0.013) | 7.13E-08 |
| **RC to LDL-C Ratio**, 10% (Friedewald) |  |  |
| Crude Model | 0.010 (0.008, 0.013) | 1.96E-16 |
| Model 1 | 0.010 (0.008, 0.012) | 1.12E-18 |
| Model 2 | 0.010 (0.008, 0.012) | 1.26E-17 |
| Model 3 | 0.003 (0.002, 0.004) | 1.14E-06 |
| **RC to LDL-C Ratio**, 10% (Sampson) |  |  |
| Crude Model | 0.018 (0.015, 0.020) | 9.02E-30 |
| Model 1 | 0.017 (0.015, 0.019) | 1.97E-31 |
| Model 2 | 0.017 (0.014, 0.019) | 3.36E-30 |
| Model 3 | 0.005 (0.003, 0.007) | 5.96E-08 |

**Notes:** Model 1 adjusted for age (continuous), gender (female and male), ethnicity (white, black, or other), education level (below high school, or high school and above), family income (poor, near poor, or non-poor), and marital status (non-married or married). Model 2 adjusted for the variables in model 1 plus smoking status (never, former and current) and HEI-2015 (quartile). Model 3 adjusted for the variables in model 2 plus BMI (continuous), SBP (continuous), DBP (continuous), eGFR level (≥ 90, 60 to 89, and < 60 ml/min per 1.73 m^2^), CVD (no or yes), DM (no or yes), statins use (no or yes), anti-Diabetic drug use (no or yes), and anti-Hypertensive drug use (no or yes). Model 4 adjusted for the variables in model 3 plus TC (continuous) and LDL-C (continuous). OR, odds ratio; CI, confidence interval; RC, remnant cholesterol; TC, total cholesterol; LDL-C, low-density lipoprotein cholesterol.


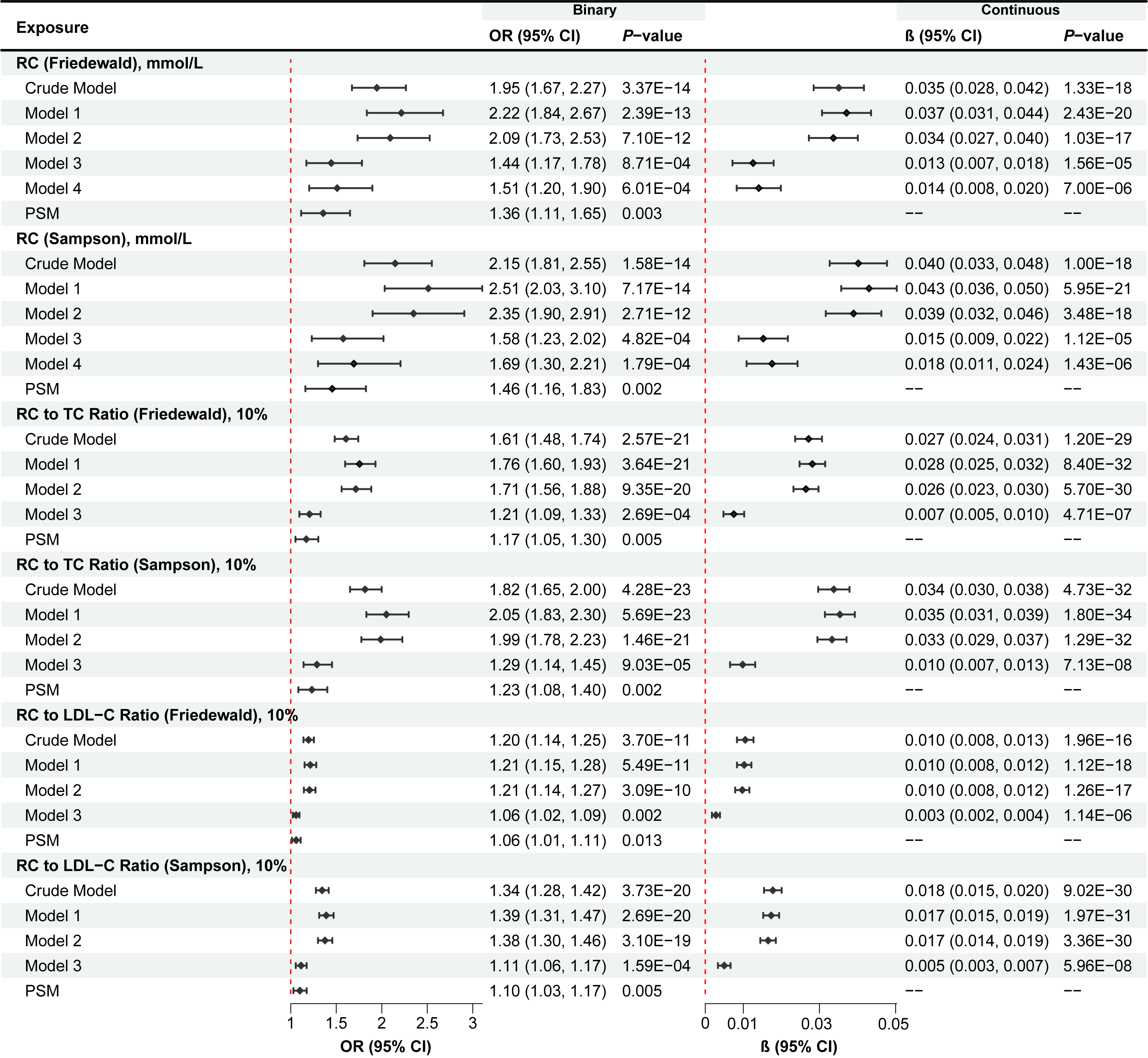


**Supplementary Figure 3. Forest Plot for** **Association of RC, RC-to-TC ratio, and RC-to-LDL-C ratio with Frailty.** The value of LDL-C was calculated using Friedewald and Sampson equation. Model 1 adjusted for age (continuous), gender (female and male), ethnicity (white, black, or other), education level (below high school, or high school and above), family income (poor, near poor, or non-poor), and marital status (non-married or married). Model 2 adjusted for the variables in model 1 plus smoking status (never, former, or current) and HEI-2015 (quartile). Model 3 adjusted for the variables in model 2 plus BMI (continuous), SBP (continuous), DBP (continuous), eGFR level (≥ 90, 60 to 89, and < 60 ml/min per 1.73 m^2^), CVD (no or yes), DM (no or yes), statins use (no or yes), anti-Diabetic drug use (no or yes), and anti-Hypertensive drug use (no or yes). Model 4 adjusted for the variables in model 3 plus TC (continuous) and LDL-C (continuous). OR, odds ratio; CI, confidence interval; RC, remnant cholesterol; TC, total cholesterol; LDL-C, low-density lipoprotein cholesterol.

**Supplementary Table 11. Instrumental Variables for RC.**

| **SNP** | **CHR** | **POS** | **Exposure** | | | **Outcome** | | | **F-statistic** |
| --- | --- | --- | --- | --- | --- | --- | --- | --- | --- |
|  |  |  | **Beta** | **SE** | ***P*-value** | **Beta** | **SE** | ***P*-value** |  |
| rs1002687 | 1 | 62963737 | 0.056 | 0.004 | 8.50E-42 | -0.002 | 0.0035 | 5.94E-01 | 171.08 |
| rs11206517 | 1 | 55526428 | 0.090 | 0.011 | 7.80E-15 | 0.019 | 0.0092 | 3.84E-02 | 60.98 |
| rs11591147 | 1 | 55505647 | -0.309 | 0.016 | 7.30E-90 | -0.026 | 0.0125 | 4.08E-02 | 390.20 |
| rs12740374 | 1 | 109817590 | -0.096 | 0.005 | 1.80E-85 | -0.005 | 0.004 | 2.04E-01 | 379.72 |
| rs1293261 | 1 | 25713234 | 0.024 | 0.004 | 7.10E-10 | -0.002 | 0.0033 | 5.97E-01 | 34.47 |
| rs472495 | 1 | 55521313 | 0.032 | 0.004 | 3.20E-14 | -0.006 | 0.0035 | 9.07E-02 | 54.97 |
| rs553427 | 1 | 234852760 | 0.029 | 0.004 | 2.80E-13 | 0.003 | 0.0033 | 4.54E-01 | 49.21 |
| rs1260326 | 2 | 27730940 | -0.038 | 0.004 | 7.10E-20 | -0.005 | 0.0034 | 1.23E-01 | 80.84 |
| rs35633876 | 2 | 20363074 | -0.024 | 0.004 | 1.20E-08 | -0.004 | 0.0033 | 2.96E-01 | 33.27 |
| rs4299376 | 2 | 44072576 | -0.047 | 0.004 | 5.40E-27 | -0.006 | 0.0035 | 9.53E-02 | 114.92 |
| rs60403635 | 2 | 21272778 | -0.074 | 0.010 | 3.70E-14 | -0.009 | 0.0082 | 2.96E-01 | 53.02 |
| rs693 | 2 | 21232195 | 0.077 | 0.004 | 5.40E-83 | -0.004 | 0.0033 | 2.25E-01 | 355.32 |
| rs9682783 | 3 | 136539689 | 0.027 | 0.005 | 4.10E-08 | 0.010 | 0.004 | 1.30E-02 | 29.26 |
| rs12916 | 5 | 74656539 | 0.058 | 0.004 | 1.40E-44 | 0.008 | 0.0034 | 1.51E-02 | 194.09 |
| rs1500188 | 5 | 122827953 | -0.024 | 0.004 | 4.90E-09 | 0.002 | 0.0033 | 5.71E-01 | 32.66 |
| rs6882345 | 5 | 156397673 | 0.041 | 0.004 | 1.90E-22 | 0.002 | 0.0034 | 6.11E-01 | 94.07 |
| rs3777411 | 6 | 160476945 | -0.049 | 0.006 | 8.50E-18 | 0.009 | 0.0047 | 6.11E-02 | 73.09 |
| rs10260606 | 7 | 44584551 | 0.034 | 0.005 | 8.20E-11 | 0.008 | 0.0043 | 6.89E-02 | 40.19 |
| rs1057558 | 7 | 1062366 | -0.025 | 0.005 | 3.00E-08 | -0.004 | 0.004 | 3.59E-01 | 26.41 |
| rs112875651 | 8 | 126506694 | -0.068 | 0.004 | 6.10E-61 | -0.003 | 0.0034 | 3.74E-01 | 258.10 |
| rs2326077 | 8 | 59385919 | -0.027 | 0.004 | 7.30E-11 | -0.004 | 0.0035 | 2.40E-01 | 38.33 |
| rs4876611 | 8 | 116671848 | 0.028 | 0.005 | 1.40E-09 | 0.005 | 0.0037 | 1.70E-01 | 37.90 |
| rs6601299 | 8 | 9184691 | 0.045 | 0.007 | 9.50E-12 | 0.014 | 0.0055 | 1.07E-02 | 44.32 |
| rs115478735 | 9 | 136151806 | 0.059 | 0.005 | 1.80E-30 | 0.007 | 0.0041 | 9.19E-02 | 123.89 |
| rs2478237 | 10 | 94804733 | -0.021 | 0.004 | 4.10E-08 | -0.001 | 0.0034 | 7.97E-01 | 25.21 |
| rs102275 | 11 | 61557803 | -0.045 | 0.004 | 1.50E-27 | -0.015 | 0.0035 | 1.35E-05 | 111.31 |
| rs59379014 | 11 | 126228000 | 0.060 | 0.008 | 8.40E-14 | -0.005 | 0.0063 | 4.76E-01 | 58.09 |
| rs964184 | 11 | 116648917 | -0.071 | 0.006 | 4.00E-34 | -0.007 | 0.0049 | 1.51E-01 | 139.63 |
| rs1169292 | 12 | 121426478 | 0.031 | 0.004 | 4.70E-12 | 0.005 | 0.0036 | 1.65E-01 | 48.48 |
| rs653178 | 12 | 112007756 | 0.025 | 0.004 | 6.60E-10 | -0.010 | 0.0033 | 1.96E-03 | 37.14 |
| rs6602911 | 13 | 114547372 | 0.024 | 0.004 | 1.90E-08 | -0.004 | 0.0034 | 2.85E-01 | 31.89 |
| rs72694391 | 14 | 24874026 | 0.022 | 0.004 | 3.50E-08 | 0.003 | 0.0033 | 3.55E-01 | 29.28 |
| rs10162642 | 15 | 58577163 | -0.043 | 0.005 | 1.50E-18 | 0.008 | 0.0041 | 6.24E-02 | 71.93 |
| rs261290 | 15 | 58678720 | -0.076 | 0.004 | 8.80E-71 | -0.006 | 0.0035 | 1.05E-01 | 308.15 |
| rs488490 | 15 | 58737661 | -0.070 | 0.006 | 1.40E-32 | 0.003 | 0.0047 | 5.65E-01 | 140.72 |
| rs247616 | 16 | 56989590 | -0.061 | 0.004 | 1.70E-43 | -0.009 | 0.0035 | 1.63E-02 | 194.07 |
| rs3794695 | 16 | 72097827 | 0.040 | 0.005 | 2.30E-15 | 0.006 | 0.0042 | 1.56E-01 | 59.59 |
| rs7202323 | 16 | 72217113 | -0.027 | 0.005 | 4.80E-08 | 0.000 | 0.0039 | 9.77E-01 | 29.70 |
| rs56325564 | 17 | 45781799 | 0.030 | 0.004 | 8.00E-14 | 0.003 | 0.0033 | 3.64E-01 | 53.45 |
| rs72631343 | 17 | 67191270 | -0.043 | 0.006 | 7.00E-13 | -0.004 | 0.005 | 4.00E-01 | 49.38 |
| rs77542162 | 17 | 67081278 | 0.149 | 0.014 | 1.30E-26 | 0.013 | 0.0111 | 2.46E-01 | 116.60 |
| rs77960347 | 18 | 47109955 | 0.136 | 0.018 | 1.20E-14 | -0.005 | 0.0145 | 7.25E-01 | 57.82 |
| rs9304381 | 18 | 47158234 | 0.031 | 0.005 | 7.70E-10 | -0.001 | 0.0043 | 7.75E-01 | 34.09 |
| rs117310449 | 19 | 45393516 | 0.178 | 0.019 | 1.30E-21 | -0.028 | 0.0151 | 6.30E-02 | 88.32 |
| rs12151108 | 19 | 11197261 | -0.153 | 0.006 | 8.49E-133 | -0.015 | 0.0052 | 3.13E-03 | 588.49 |
| rs150057262 | 19 | 19320825 | -0.110 | 0.020 | 2.40E-08 | -0.005 | 0.0162 | 7.66E-01 | 30.09 |
| rs2738447 | 19 | 11227480 | 0.033 | 0.004 | 2.20E-14 | 0.006 | 0.0034 | 5.88E-02 | 60.62 |
| rs41289512 | 19 | 45351516 | 0.080 | 0.010 | 4.10E-16 | 0.001 | 0.0082 | 8.93E-01 | 62.63 |
| rs41290120 | 19 | 45382675 | -0.371 | 0.010 | 1.00E-200 | -0.020 | 0.0078 | 9.27E-03 | 1519.12 |
| rs58542926 | 19 | 19379549 | -0.118 | 0.008 | 3.10E-51 | -0.004 | 0.0062 | 5.04E-01 | 226.95 |
| rs1883711 | 20 | 39179822 | 0.110 | 0.012 | 4.40E-21 | 0.009 | 0.0095 | 3.36E-01 | 83.98 |
| rs9616847 | 22 | 50868669 | 0.025 | 0.004 | 7.30E-09 | -0.002 | 0.0034 | 4.85E-01 | 35.10 |

**Notes**: SNP, single nucleotide polymorphism; CHR, chromosome; POS, Position; SE, standard error.

**Supplementary Table 12. Instrumental Variables for Frailty Index**

| **SNP** | **CHR** | **POS** | **Exposure** | | | **Outcome** | | | **F-statistic** |
| --- | --- | --- | --- | --- | --- | --- | --- | --- | --- |
|  |  |  | **Beta** | **SE** | ***P*-value** | **Beta** | **SE** | ***P*-value** |  |
| rs12739243 | 1 | 210302043 | -0.024 | 0.004 | 1.28E-09 | -0.027 | 0.005 | 0.21 | 36.60 |
| rs4952693 | 2 | 44151808 | -0.019 | 0.003 | 1.47E-08 | -0.011 | 0.004 | 0.47 | 32.56 |
| rs2071207 | 3 | 50159844 | -0.019 | 0.003 | 1.47E-08 | 0.020 | 0.004 | 0.14 | 32.11 |
| rs583514 | 3 | 173114167 | 0.020 | 0.003 | 1.65E-09 | -0.011 | 0.004 | 0.450 | 36.36 |
| rs82334 | 4 | 3225371 | -0.022 | 0.004 | 3.13E-10 | -0.016 | 0.004 | 0.35 | 40.59 |
| rs1363103 | 5 | 103917837 | -0.019 | 0.003 | 2.23E-08 | -0.025 | 0.004 | 0.12 | 31.56 |
| rs374943348 | 6 | 32619856 | 0.027 | 0.004 | 3.23E-12 | -0.027 | 0.005 | 0.25 | 49.36 |
| rs555911977 | 6 | 32535454 | 0.034 | 0.005 | 2.10E-13 | -0.102 | 0.006 | 2.6E-05 | 54.63 |
| rs9275160 | 6 | 32652620 | 0.038 | 0.004 | 7.18E-28 | -0.059 | 0.004 | 6.3E-04 | 119.12 |
| rs2396766 | 7 | 114318071 | 0.020 | 0.003 | 1.22E-09 | -0.003 | 0.004 | 0.79 | 37.10 |
| rs56299474 | 8 | 21992804 | 0.024 | 0.004 | 3.94E-08 | -0.026 | 0.005 | 0.29 | 30.00 |
| rs4146140 | 10 | 61885362 | -0.020 | 0.003 | 6.83E-09 | 0.014 | 0.004 | 0.27 | 33.91 |
| rs10891490 | 11 | 112885527 | -0.019 | 0.003 | 2.00E-08 | -0.015 | 0.004 | 0.40 | 30.57 |
| rs17612102 | 15 | 52264094 | 0.019 | 0.003 | 2.85E-08 | 0.008 | 0.004 | 0.67 | 30.25 |
| rs3959554 | 15 | 41443924 | 0.019 | 0.003 | 1.74E-08 | -0.009 | 0.004 | 0.49 | 30.90 |
| rs8089807 | 18 | 39322639 | -0.025 | 0.004 | 6.50E-09 | -0.005 | 0.005 | 0.68 | 33.26 |

**Notes**: SNP, single nucleotide polymorphism; CHR, chromosome; POS, Position; SE, standard error.

**Supplementary Table 13. Instrumental Variables for Fried Frailty Phenotype.**

| **SNP** | **CHR** | **POS** | **Exposure** | | | **Outcome** | | | **F-statistic** |
| --- | --- | --- | --- | --- | --- | --- | --- | --- | --- |
|  |  |  | **Beta** | **SE** | ***P*-value** | **Beta** | **SE** | ***P*-value** |  |
| rs10053447 | 5 | 153105318 | 0.0124 | 0.0019 | 3.10E-11 | -0.0056 | 0.0042 | 2.00E-01 | 43.78 |
| rs1048027 | 6 | 32609169 | -0.0106 | 0.0019 | 3.80E-08 | 0.0085 | 0.0043 | 5.00E-02 | 30.43 |
| rs10828258 | 10 | 21929734 | -0.0142 | 0.0020 | 7.50E-13 | 0.0089 | 0.0044 | 6.20E-02 | 51.87 |
| rs10986468 | 9 | 127624679 | 0.0115 | 0.0018 | 4.10E-10 | -0.0015 | 0.0041 | 7.90E-01 | 38.91 |
| rs11130207 | 3 | 49611666 | 0.0153 | 0.0025 | 5.30E-10 | 0.0019 | 0.0056 | 8.70E-01 | 38.00 |
| rs11150602 | 16 | 30993823 | 0.0107 | 0.0019 | 2.00E-08 | 0.0043 | 0.0043 | 2.90E-01 | 31.69 |
| rs11660938 | 18 | 50812736 | -0.0105 | 0.0019 | 1.50E-08 | 0.0022 | 0.0042 | 5.30E-01 | 31.82 |
| rs11689546 | 2 | 25130451 | -0.0117 | 0.0019 | 2.90E-10 | 0.0055 | 0.0042 | 1.40E-01 | 39.43 |
| rs12601919 | 17 | 65825374 | -0.0129 | 0.0023 | 3.20E-08 | -0.0172 | 0.0053 | 4.60E-04 | 30.21 |
| rs12712072 | 2 | 100797912 | 0.0146 | 0.0022 | 1.60E-11 | -0.0027 | 0.0048 | 4.70E-01 | 45.80 |
| rs12992177 | 2 | 232312968 | -0.0165 | 0.0030 | 4.00E-08 | -0.0012 | 0.0067 | 8.80E-01 | 30.23 |
| rs13107325 | 4 | 103188709 | -0.0310 | 0.0035 | 7.60E-19 | 0.0108 | 0.0078 | 1.30E-01 | 78.55 |
| rs1421085 | 16 | 53800954 | -0.0130 | 0.0019 | 3.00E-12 | 0.0127 | 0.0042 | 4.80E-03 | 48.22 |
| rs17707300 | 16 | 28593347 | -0.0120 | 0.0019 | 2.50E-10 | -0.0002 | 0.0042 | 8.70E-01 | 40.35 |
| rs17716502 | 8 | 116659731 | 0.0131 | 0.0023 | 9.00E-09 | 0.0193 | 0.0051 | 1.60E-04 | 32.94 |
| rs2044169 | 17 | 2394896 | -0.0111 | 0.0020 | 3.90E-08 | -0.0003 | 0.0045 | 7.70E-01 | 30.33 |
| rs2287234 | 2 | 51258887 | 0.0106 | 0.0018 | 1.10E-08 | -0.0057 | 0.0041 | 1.20E-01 | 32.99 |
| rs2847308 | 11 | 57498232 | 0.0121 | 0.0019 | 5.90E-10 | -0.0051 | 0.0044 | 1.60E-01 | 38.64 |
| rs28509789 | 15 | 75499995 | 0.0119 | 0.0021 | 3.30E-08 | 0.0044 | 0.0048 | 3.10E-01 | 30.54 |
| rs2857597 | 6 | 31585000 | -0.0120 | 0.0020 | 3.40E-09 | -0.0038 | 0.0046 | 3.20E-01 | 34.73 |
| rs303762 | 18 | 21082317 | -0.0106 | 0.0019 | 3.80E-08 | -0.0032 | 0.0043 | 5.10E-01 | 30.44 |
| rs362307 | 4 | 3241845 | -0.0287 | 0.0035 | 1.50E-16 | -0.0173 | 0.0079 | 1.90E-02 | 67.70 |
| rs3821269 | 2 | 70698590 | 0.0110 | 0.0018 | 2.50E-09 | 0.0033 | 0.0041 | 4.20E-01 | 35.70 |
| rs4457304 | 8 | 110028183 | 0.0103 | 0.0018 | 2.00E-08 | -0.0110 | 0.0041 | 1.50E-02 | 31.71 |
| rs4549685 | 7 | 39326478 | 0.0109 | 0.0019 | 1.80E-08 | -0.0042 | 0.0044 | 2.40E-01 | 31.58 |
| rs62082234 | 18 | 22691038 | -0.0105 | 0.0019 | 4.10E-08 | -0.0125 | 0.0043 | 2.00E-03 | 30.25 |
| rs62444907 | 7 | 2083267 | 0.0186 | 0.0025 | 3.10E-13 | -0.0089 | 0.0057 | 7.90E-02 | 53.50 |
| rs660010 | 18 | 53310969 | 0.0174 | 0.0026 | 2.30E-11 | 0.0024 | 0.0059 | 6.40E-01 | 44.43 |
| rs6751993 | 2 | 635864 | -0.0145 | 0.0025 | 2.50E-09 | 0.0018 | 0.0055 | 8.30E-01 | 35.00 |
| rs724701 | 14 | 47236264 | 0.0121 | 0.0021 | 1.00E-08 | -0.0127 | 0.0048 | 1.10E-02 | 32.20 |
| rs72709800 | 9 | 25513013 | -0.0208 | 0.0034 | 8.80E-10 | -0.0007 | 0.0076 | 8.90E-01 | 37.56 |
| rs7703746 | 5 | 103907337 | 0.0132 | 0.0018 | 6.40E-13 | 0.0068 | 0.0041 | 8.60E-02 | 51.55 |
| rs79659409 | 2 | 60607636 | 0.0167 | 0.0029 | 5.70E-09 | -0.0013 | 0.0065 | 9.70E-01 | 33.78 |
| rs80032406 | 11 | 74272582 | -0.0126 | 0.0021 | 1.30E-09 | -0.0004 | 0.0047 | 7.00E-01 | 36.49 |
| rs8044920 | 16 | 69838676 | -0.0107 | 0.0019 | 1.60E-08 | 0.0015 | 0.0042 | 8.50E-01 | 31.96 |
| rs9953231 | 18 | 35150526 | -0.0122 | 0.0022 | 3.00E-08 | -0.0012 | 0.0049 | 6.80E-01 | 31.05 |

**Notes**: SNP, single nucleotide polymorphism; CHR, chromosome; POS, Position; SE, standard error.

**Supplementary Table 14. Bidirectional MR** **Analyses for the Association between RC and Fried Frailty Phenotype.**

| **Methods** | **Effect of RC on Frailty Phenotype** | | **Effect of Frailty Phenotype on RC** | |
| --- | --- | --- | --- | --- |
|  | **OR (95% CI)** | ***P*-value** | **β (95% CI)** | ***P*-value** |
| IVW | 0.99 (0.98, 1.01) | 0.259 | -0.05 (-0.24, 0.13) | 0.572 |
| MR-Egger | 0.98 (0.96, 1.00) | 0.095 | 0.10 (-0.68, 0.87) | 0.806 |
| Weighted median | 0.99 (0.97, 1.00) | 0.105 | -0.10 (-0.28, 0.08) | 0.806 |
| Simple median | 0.99 (0.97, 1.01) | 0.279 | -0.03 (-0.22, 0.16) | 0.742 |
| MR-PRESSO | 0.99 (0.98, 1.01) | 0.265 | -0.05 (-0.24, 0.13) | 0.575 |


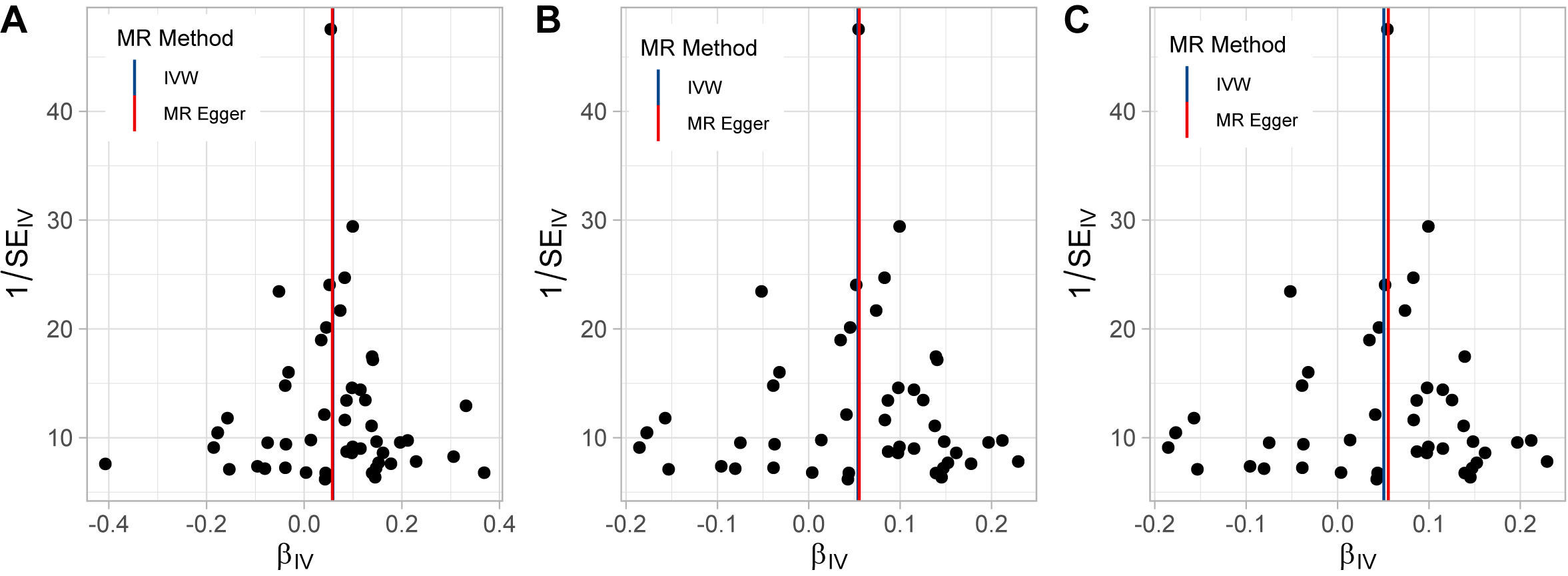


**Supplementary Figure 4. Funnel plot. (A) Raw; (B) Removing rs653178, rs9682783, rs102275, and rs6601299; (C) Removing rs12916, rs4876611, rs653178, rs9682783, rs102275, and rs6601299.**

**Supplementary Table 15. MR Results for Effect of RC on FI Removing four SNPs.**

| **Methods** | **β (95% CI)** | ***P*-value** | **Heterogeneity test** | | |
| --- | --- | --- | --- | --- | --- |
|  |  |  | ***Q*** | **I^2^** | ***P*-value** |
| IVW | 0.054 (0.032, 0.076) | 1.97E-06 | 61.26 | 24.91% | 0.065 |
| MR-Egger | 0.055 (0.019, 0.091) | 4.40E-03 | 61.25 | 26.53% | 0.054 |
| Weighted median | 0.061 (0.030, 0.092) | 1.13E-04 | -- | -- | -- |
| Simple median | 0.107 (0.055, 0.159) | 2.08E-04 | -- | -- | -- |

**Notes:** Four SNPs, including rs653178, rs9682783, rs102275, and rs6601299, were removed.

**Supplementary Table 16. MR Results for Effect of RC on FI Removing six SNPs.**

| **Methods** | **β (95% CI)** | **P-value** | **Heterogeneity test** | | |
| --- | --- | --- | --- | --- | --- |
|  |  |  | **Q** | **I^2^** | ***P*-value** |
| IVW | 0.051 (0.028, 0.073) | 1.02E-05 | 58.05 | 24.21% | 0.076 |
| MR-Egger | 0.056 (0.019, 0.092) | 4.28E-03 | 57.89 | 25.73% | 0.064 |
| Weighted median | 0.059 (0.026, 0.091) | 4.28E-04 | -- | -- | -- |
| Simple median | 0.099 (0.044, 0.153) | 9.32E-04 | -- | -- | -- |

**Notes:** Six SNPs, including rs12916, rs4876611, rs653178, rs9682783, rs102275, and rs6601299, were removed.
