# Supplementary material for: Association of remnant cholesterol with frailty: findings from observational and Mendelian randomization analyses
Source: Lipids Health Dis. 2023 Aug 3;22:115. doi: 10.1186/s12944-023-01882-4 (PMC10399004; doi:10.1186/s12944-023-01882-4)
Supplement: Supplementary file 2 — Additional file 2. [file 12944_2023_1882_MOESM2_ESM.pdf]

EditorBar Language Editing
No. 35, Tsinghua East Road, Beijing, China 100083
Email: runse@editorbar.com Phone: +86-10-5620-8614

CERTIFICATE OF LANGUAGE EDITING

The English writing of the following manuscript was carefully edited by a native English speaker.

Manuscript Information

ID	AE202307120159
Editing date	2023-07-16
Title	Association of Remnant Cholesterol with Frailty: Findings from Observational and Mendelian Randomization Analyses
Corresponding author	Yuanlong Hu
Language writing before editing	□ Very poor □ Poor ■ Fair □ Good □ Very good □ Excellent
Recommendation after language editing	■ Submitting to target journal directly<br>□ Submitting to target journal after minor revision<br>□ Re-editing required after major revision<br>□ Not suitable for publication
Overview comments	Dear Authors, I am genuinely impressed by the exceptional quality of your research and the diligence displayed in the manuscript's composition. Your study will undeniably make a significant impact on the scientific community. My revisions were intended to further elevate your work, refining the language for greater clarity, and ensuring your findings and insights are effectively and impactfully communicated. In doing so, sentences were restructured and word usage adjusted as needed. These enhancements will augment your work's reach and resonance. I applaud your dedication and thank you for contributing your significant findings to the scientific community. Kindest regards, Phoebe C., Editor

Edited by

Phoebe C.
Senior editor
University of Texas
Language Editing

Certificate Issued by

Dr. Jason Qee

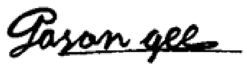

Editor in Chief
Editorbar Language Editing, Beijing, China
runse@editorbar.com www.editorbar.com

Certificate link: www.editorbar.com/order/cert/AE202307120159
